# Large-scale phylogenomics reveals ancient introgression in Asian *Hepatica* and new insights into the origin of the insular endemic *Hepatica maxima*

**DOI:** 10.1038/s41598-020-73397-2

**Published:** 2020-10-01

**Authors:** Seongjun Park, SeonJoo Park

**Affiliations:** 1 Institute of Natural Science, Yeungnam University, Gyeongsan, Gyeongbuk 38541 South Korea; 2 Department of Life Sciences, Yeungnam University, Gyeongsan, Gyeongbuk 38541 South Korea

**Keywords:** Phylogenetics, Evolution, Plant sciences, Plant evolution

## Abstract

*Hepatica maxima* is native to Ulleungdo, which is one of the oceanic islands in Korea, and it likely originated via anagenetic speciation from the Korean mainland species *H. asiatica*. However, the relationships among the Asian lineages remain unresolved. Phylogenomics based on plant genomes can provide new insights into the evolutionary history of plants. We first generated plastid, mitochondrial and transcriptome sequences of the insular endemic species *H. maxima*. Using the genomic data for *H. maxima*, we obtained a phylogenomic dataset consisting of 76 plastid, 37 mitochondrial and 413 nuclear genes from Asian *Hepatica* and two outgroups. Coalescent- and concatenation-based methods revealed cytonuclear and organellar discordance in the lineage. The presence of gynodioecy with cytoplasmic male sterility in Asian *Hepatica* suggests that the discordance is correlated with potential disruption of linkage disequilibrium between the organellar genomes. Species network analyses revealed a deep history of hybridization and introgression in Asian *Hepatica.* We discovered that ancient and recent introgression events occurred throughout the evolutionary history of the insular endemic species *H. maxima*. The introgression may serve as an important source of genetic variation to facilitate adaptation to the Ulleungdo environment.

## Introduction

Oceanic islands are ideal evolutionary laboratories for investigating the processes of plant speciation. Two modes of speciation, cladogenesis and anagenesis, are observed on oceanic islands^1^. During cladogenesis, an initial population splits into two lineages through isolation, which leads to genetic and morphological variation between the lineages^1^. During anagenesis, continental progenitor populations slowly transform via genetic and morphological variation through time without a lineage-splitting event^1–3^. Oceanic islands are usually small land masses that are well isolated from the mainland and have high levels of endemism^1^. Ulleungdo is an oceanic island with a total area of 73 km^2^, and it is located 137 km east of the Korean Peninsula^4^. It is home to a small flora of only 33 endemic plant species^2,5^. However, Ulleungdo provides great opportunities for understanding the two principal modes of speciation and shows a higher level of endemism derived via anagenesis (88%) than cladogenesis (12%)^2^. Multiple studies have focused on understanding the genetic variation between endemic species on Ulleungdo and their continental progenitors^6–9^.

The genus *Hepatica* (Ranunculaceae) is distributed in temperate zones of the Northern Hemisphere^10^ and shows high diversification in eastern Asia characterized by four species (*H. asiatica*, *H. henryi*, *H. insularis*, and *H. maxima*) and two varieties (*H. nobilis* var. *japonica* and *H. nobilis* var. *pubescens*). *Hepatica maxima* (Nakai) Nakai is endemic to Ulleungdo and likely originated via anagenetic speciation^11^ from the Korean mainland species *H. asiatica*^12^. *Hepatica maxima* exhibits “insular gigantism”, with increased leaf, bract and seed sizes compared to those of the putative progenitor^13^. In addition, *H. maxima* is distinguished by glabrous achenes and leaf cuticles^13,14^. A previous study estimated the phylogenetic relationships within the genus *Hepatica* using nuclear (ITS) and plastid (*atpB-rbcL* spacer and *trnL-F* intron/spacer) markers and amplified fragment length polymorphism (AFLP) data^12^, however the relationships within the genus *Hepatica* remain unresolved. Resolving interspecific relationships within *Hepatica* is important for reconstructing the evolutionary history of *H. maxima*.

Plant cells have three distinct genomes (nuclear, plastid and mitochondrial genomes), with each providing valuable resources for studying plant evolution. For example, organellar genomic information provides a better understanding of plant evolution^15,16^. In particular, variation in organellar genomes can contribute to plant adaptation^17^. Plastid and mitochondrial genomes (the plastome and mitogenome, respectively) are generally inherited maternally in higher plants^18,19^, although several exceptions are observed^20,21^. Compared with organellar genomes, the nuclear genome is biparentally inherited and presents better resolution among closely related species with high mutation rates^22,23^. Nuclear genes provide powerful opportunities to address adaptation to environmental changes^24–27^.

Genome-scale data improve the understanding of the origin of species and lineages that undergo rapid evolutionary radiation. Thus, comparative analyses of nuclear, plastid, and mitochondrial genomes are required for understanding the origin of the insular endemic species *H. maxima*. The development of next-generation sequencing (NGS) techniques and methods^28,29^ enables us to assemble complete plastomes and mitogenomes as well as nuclear transcriptomes for plants. In addition, third-generation sequencing platforms such as those of Pacific Biosciences (PacBio) and Oxford Nanopore Technologies (ONT)^30^ facilitate the assembly of large and complex genomes. However, while one plastome sequence is available for *Hepatica*, data on the mitochondrial and nuclear genomes in this genus is lacking.

In this study, we first generated the plastid and mitochondrial genomes and nuclear transcriptome of the insular endemic species *H. maxima*. To reconstruct a genome-scale phylogeny for the Asian *Hepatica*, we sequenced complete plastome and draft mitogenome sequences from six additional taxa, including four *Hepatica* as well as *Anemone narcissiflora* and *Pulsatilla koreana*, as well as six nuclear transcriptomes. The patterns of incongruence between organellar and nuclear genomes and within the organellar genomes were identified. Additional comparative genomic and phylogenomic analyses provide new insights into organellar genome evolution within tribe Anemoneae and the evolutionary origin of the insular endemic species *H. maxima*.

## Results

### Plastid and mitochondrial genomes

**Figure 1:**
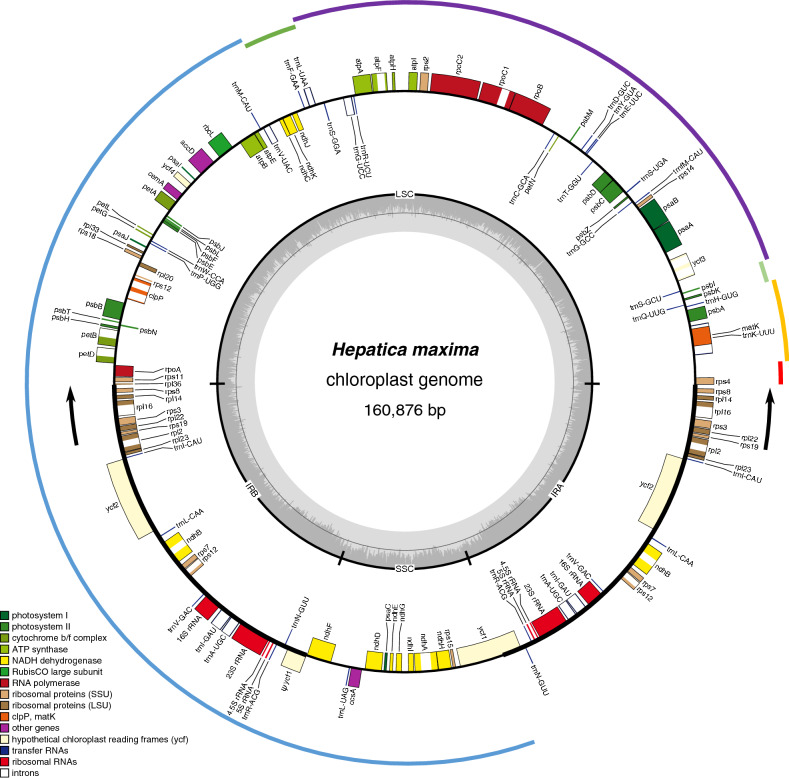
Map of the plastid genome of *Hepatica maxima*. Genes on the inside and outside of each map are transcribed in the clockwise and counterclockwise directions, respectively. The thick lines on the plastid map indicate the inverted repeats (IRa and IRb) that separate the genome into large and small single-copy regions. Ψ denotes a pseudogene. Black arrows indicate expansion events. Colored arcs on the outside of the map correspond to the locally collinear blocks inferred by Mauve (see Supplementary Fig. S3). The figures were constructed in OGDRAW v1.3.1 (https://chlorobox.mpimp-golm.mpg.de/OGDraw.html) and InkScape v0.92.2 (https://inkscape.org).

The organellar genomes of the insular endemic species *H. maxima* were sequenced on the Illumina and Oxford platforms and completed by Velvet and Spades assemblies with optimized parameter values. The plastome of *H. maxima* was 160,876 bp in length (≈ 1250 × depth of coverage), with a pair of inverted repeats (IRs) (IR_A_ and IR_B_) of 31,097 bp separated by large and small single-copy (LSC and SSC) regions of 80,998 and 17,684 bp, respectively (Fig. 1 and Table 1). The plastome contained 110 different genes, including 76 protein-coding genes, 29 tRNA genes, and four rRNA genes (Table 1). Three protein-coding genes, namely, translation initiation factor A (*infA*), ribosomal protein subunit L32 (*rpl32*), and ribosomal protein subunit S16 (*rps16*), and one tRNA gene, *trnT-UGU*, were absent. The plastome had an expanded IR with the IR_A_/LSC boundary shifted from *rps19* into *rps8* (Fig. 1).

**Table 1:** Summary of *Hepatica maxima* plastid and mitochondrial genomes.

	Plastome	Mitogenome
Genome size (bp)	160,876	1,122,546
Protein coding genes	76	39
rRNA genes	4	3
tRNA genes	29	17 (6)
Introns		
*cis*-spliced	19	18
*trans*-spliced	1	5
GC content (%)	37.8	46.2
Repeat content (%)	0.48	6.94
Coding content (%)	50.37	3.28

**Figure 2:**
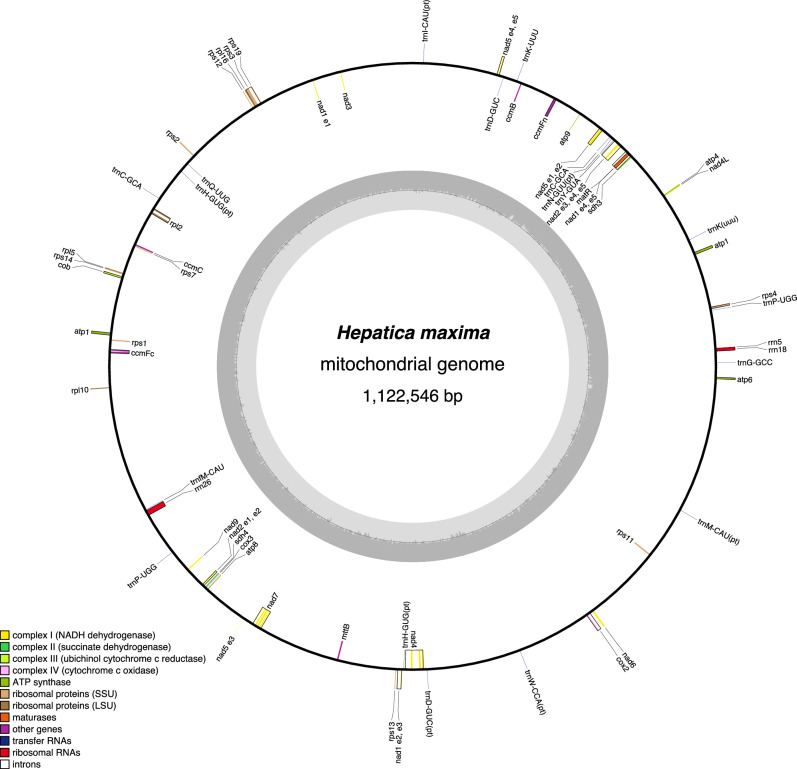
Map of the mitochondrial genome of *Hepatica maxima*. Genes on the inside and outside of each map are transcribed in the clockwise and counterclockwise directions, respectively. The figures were constructed in OGDRAW v1.3.1 (https://chlorobox.mpimp-golm.mpg.de/OGDraw.html).

The *H. maxima* mitogenome was assembled into a 1,122,546 bp circular molecule (≈ 110 × depth of coverage) and contained 39 protein-coding genes, 3 rRNA genes, 11 tRNA genes, 18 *cis*-spliced introns, and 5 *trans*-spliced introns (Table 1 and Fig. 2). Two ribosomal protein genes, *rps10* and *rps14,* were absent or appeared to be pseudogenes. The mitogenome contained 77,887 bp of repetitive DNA, ranging from 31 to 19,922 bp in length. The mitogenome possessed 23 fragments of plastid-derived sequences ranging from 51 bp to 7,104 bp in length (Supplementary Table S1) and covering 1.54% of the genome. PREP-Mt predicted 687 putative C-to-U RNA editing sites in the 39 mitochondrial protein-coding genes (Supplementary Table S2). Eighteen open reading frames (ORFs) at least 150 bp in length were identified that contain small fragments (> 30 bp) of one or more mitochondrial genes (Supplementary Table S3). These ORFs contained small fragments of the nine mitochondrial genes *atp1*, *atp6*, *atp9*, *cox1*, *ccmFc*, *nad6*, *rps19*, *rps4*, and *rrn26*. Five of these ORFs (*orf297*, *orf296*, *orf194*, *orf138*, and *orf51*) were predicted to encode one or two transmembrane helices (Supplementary Fig. S1). *orf296* was immediately downstream from a repeat (929,551–929,873) that overlapped with the *atp1* gene, thus, the first 27 bp of *orf296* and *atp1* was identical (Supplementary Fig. S2). Observation of the corrected long reads supports a putative rearrangement at the repeats (Supplementary Fig. S2).

In addition, we generated raw paired-end (PE) Illumina reads that enabled de novo assembly of the complete plastome and draft mitogenome for the four *Hepatica* species and two related species *A. narcissiflora* and *P. koreana* from tribe Anemoneae. The additional *Hepatica* plastome sizes ranged from 159.8 to 160.9 kb, and the organization and gene/intron content were consistent with the results for *H. maxima* (Supplementary Table S4). The plastomes of *A. narcissiflora* and *P. koreana* showed minimal variation in genome size, IR boundary shifts, and GC content in comparison to those in the genus *Hepatica* (Supplementary Table S4). However, the plastome of *A. narcissiflora* lacked the *rps16* intron, and *P. koreana* contained an intact *rps16* gene in its plastome. For all seven taxa, 76 plastid-encoded genes were obtained from the assembled plastomes. Using BLAST searches between mitochondrial gene sequences from *H. maxima* and draft mitogenomes, 37 mitochondrion-encoded genes were obtained for all species (Supplementary Table S5).

### Genomic changes in plastid and mitochondrial genome evolution

**Figure 3:**
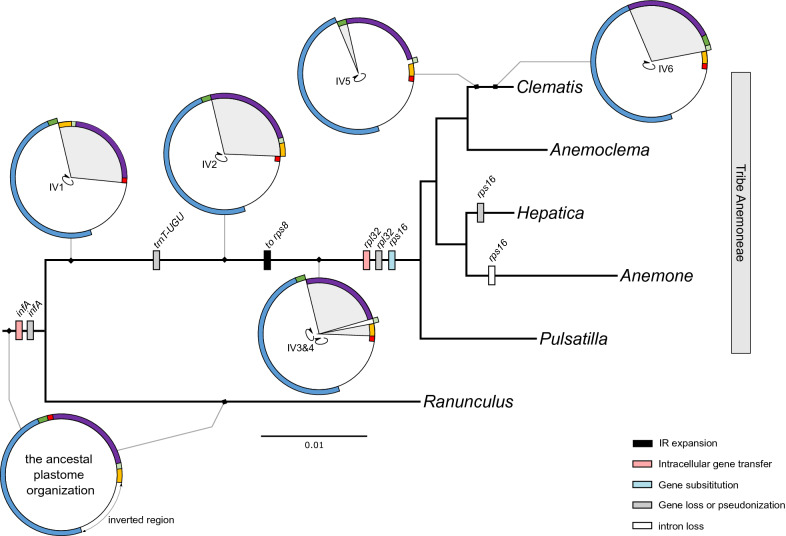
Summary of plastome rearrangement and intracellular gene transfer events in tribe Anemoneae with gene/intron loss events. The colors on the circular maps correspond to the locally collinear blocks inferred by Mauve (see Fig. 1 and Supplementary Fig. S3). IV; inversion. The figures were constructed in InkScape v0.92.2 (https://inkscape.org).

A Mauve alignment between the tribe Anemoneae and the outgroup (*Ranunculus macranthus*) identified six locally collinear blocks (LCBs) with five breakpoints that occurred at *rps16-trnQ*, *trnS-trnG*, *trnG-rps4*, *rps4-trnT*, and *ndhC-trnV* (Supplementary Fig. S3). An inferred plastome rearrangement model suggests that tribe Anemoneae has experienced six inversions (Fig. 3). The four inversion events (IV1 to IV4) occurred in the common ancestor of tribe Anemoneae, and two inversion events (IV5 and IV6) are specific to *Clematis*. The IR expansion is a synapomorphic event in tribe Anemoneae. A comparative analysis of the gene and intron contents revealed that losses of the plastid *infA*, *rpl32* and *trnT-UGU* genes are shared by all analyzed species in the tribe Anemoneae, whereas the loss of *rps16* is unique to the *Hepatica* plastome (Fig. 3).

**Figure 4:**
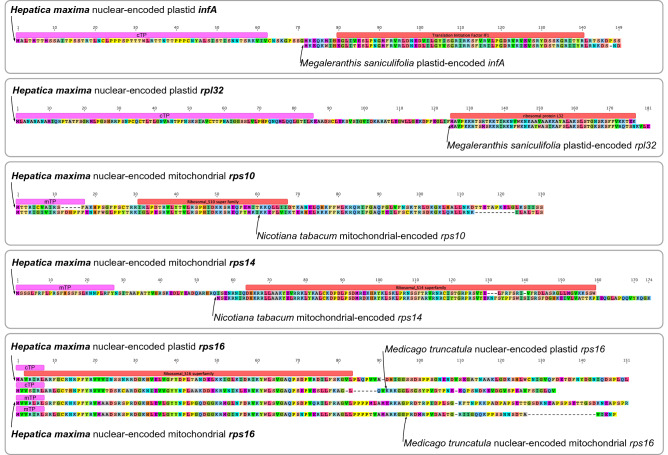
Amino acid sequence alignments of nuclear-encoded organellar genes in *Hepatica maxima* and their organellar copies in related species. Red boxes indicate the conserved domains, and pink boxes in the N-terminus indicate a transit peptide. The figures were constructed in Geneious R7 v7.1.8 (https://www.geneious.com).

We identified putative losses of three plastid (*infA*, *rpl32*, and *rps16*) and two mitochondrial (*rps10* and *rps14*) genes in the *H. maxima* organellar genomes. To examine functional transfers to the nucleus, blastn searches against the *H. maxima* transcriptome with the five organellar genes of related species were performed. All predicted ORFs of the transcripts acquired novel targeting sequences and conserved domains (Fig. 4 and Supplementary Table S6). Six additional transcriptomes provided strong evidence for the functional transfer of five organellar genes into the nucleus within tribe Anemoneae (Supplementary Table S6). In particular, we found a transcript for the nuclear-encoded plastid *rps16* gene in all additional transcriptomes, although the plastid-encoded *rps16* gene has been lost in the *Hepatica* lineage (Supplementary Table S6).

### Identification of nuclear orthologs

To enable comparisons of the nuclear genes from *Hepatica*, we further sequenced the transcriptomes of the five *Hepatica* species and two outgroups, *A. narcissiflora* and *P. koreana*. We assembled the transcriptomes de novo using Trinity, excluded a transcript that included structural and base errors, and clustered transcripts with a high level of sequence similarity (Supplementary Table S7). We identified candidate coding regions within the clustered transcripts, and extracted complete ORFs (Supplementary Table S7). A total of 69,294 putative ORFs from seven species were assigned to 11,456 orthogroups by OrthoFinder (Supplementary Table S7). A total of 1428 orthogroups were shared across all seven analyzed species, and we obtained 510 single-copy orthologs for further analysis (Supplementary Table S7). Genes showing high degrees of misleading signals as indicated by the four parameters were filtered by TreSpEx (Supplementary Table S8), resulting in 413 single-copy orthologs.

### Phylogenomic reconstructions and divergence times

All analyzed *Hepatica* species and the two related species *A. narcissiflora* and *P. koreana* shared 76 plastid-encoded, 37 mitochondrial-encoded, and 413 nuclear single-copy genes. To examine the origin of *H. maxima*, we performed phylogenomic analyses using two coalescent- and concatenation-based approaches.

**Figure 5:**
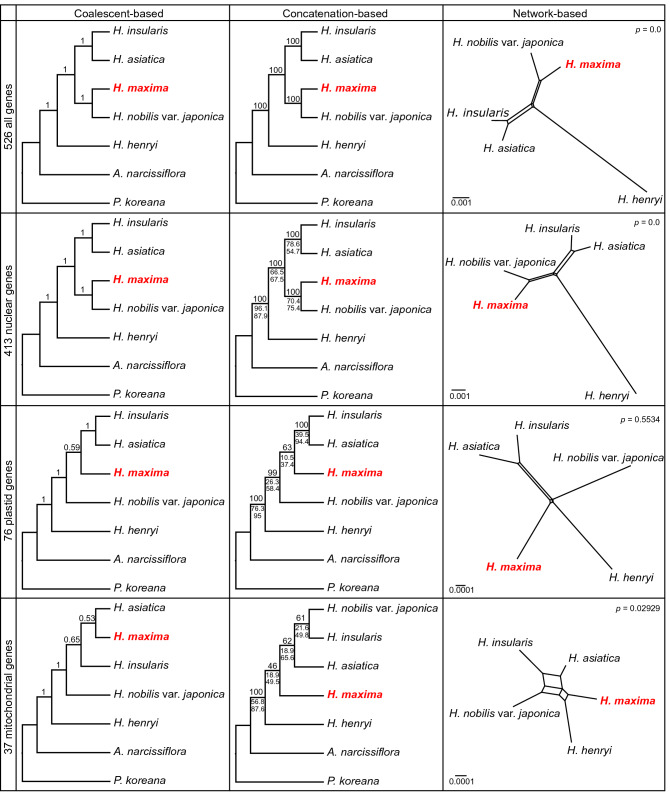
Phylogenomic trees of the nuclear, plastid, and mitochondrial genes inferred with ASTRAL-III, IQ-TREE2, and SplitsTree4. Local posterior probability and maximum likelihood bootstrap support values are shown above the branches on each cladogram. Numbers below the branches of maximum likelihood trees represent gene and site concordance factors, sequentially. The figures were constructed in ASTRAL v5.7.1 (https://github.com/smirarab/ASTRAL), IQ-TREE v2.0 (https://www.iqtree.org/), SplitsTree v4.15.1 (https://www.splitstree.org/) and modified in InkScape v0.92.2 (https://inkscape.org).

The topologies of the Accurate Species TRee ALgorithm (ASTRAL) and maximum likelihood (ML) supermatrix based on 526 genes were identical, with high support according to local posterior probability (LPP = 1.00) and bootstrap (BS = 100) values (Fig. 5). The short internal branches in the ML trees suggested rapid diversification in the *Hepatica* lineage (Supplementary Fig. S4). The analyses showed that *H. henryi* was basal to the rest of *Hepatica* with strong bootstrap support (LPP = 1.00 and BS = 100). *Hepatica maxima* and *H. nobilis* var. *japonica* as well as *H. asiatica* and *H. insularis* grouped together with high support values. However, the placement of *H*. *maxima* varied among the nuclear, plastid and mitochondrial ASTRAL and concatenated ML trees (Fig. 5). Both nuclear analyses robustly supported *H. maxima* as sister to *H. nobilis* var*. japonica* (LPP = 1.00, BS = 100, and gCF = 70.4), although the two organellar datasets were not consistent with the nuclear phylogeny. For example, the plastid ASTRAL and concatenated trees were consistent and placed *H. maxima* sister to *H. asiatica* and *H. insularis* with low support values (LPP = 0.59, BS = 63, and gCF = 10.5, Fig. 5). The mitochondrial ASTRAL tree revealed *H. maxima* as sister to *H. asiatica*, whereas the mitochondrial concatenated tree showed *H. maxima* as sister to the remaining three *Hepatica* species with weak support values (LPP = 0.53 and BS = 46, Fig. 5). The plastid and mitochondrial concordance factors were low for the four species, suggesting a high degree of conflict among the gene trees (Fig. 5). Among the organellar gene trees, we only found five plastid (6.6% of 76 plastid genes) and two mitochondrial gene trees (5.4% of 37 mitochondrial genes) that were identical to the nuclear tree, which supported *H. maxima* as sister to *H*. *nobilis* var. *japonica*. All four split networks showed reticulate topologies, although the pairwise homoplasy index (PHI) test identified recombination signals in the supermatrix, nuclear and mitochondrial datasets (Fig. 5). The ML analysis of nucleotides after removing recombination sites by Gubbins supported the gene tree topology, which was identical to the ASTRAL and supermatrix phylogenies.

**Figure 6:**
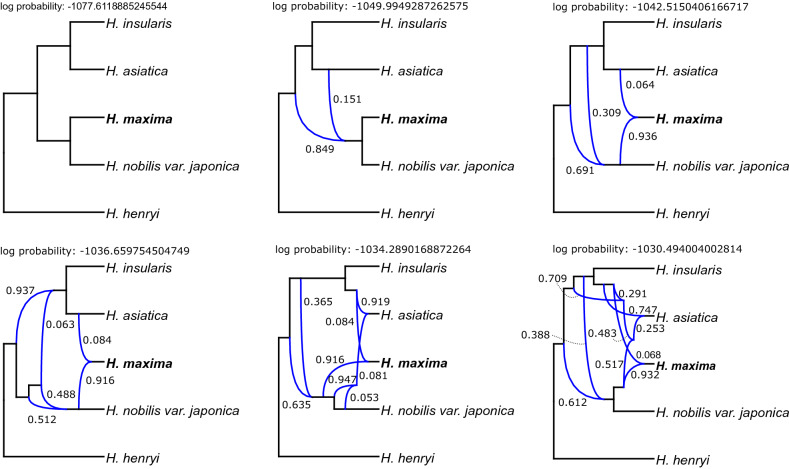
Phylogenetic networks inferred by PhyloNet using maximum likelihood under zero to six reticulation models. Blue branches indicate lineages involved in reticulated histories, and numerical values are the inheritance probabilities for each reticulation. The figures were constructed in PhyloNet (https://bioinfocs.rice.edu/phylonet) and modified in InkScape v0.92.2 (https://inkscape.org).

**Figure 7:**
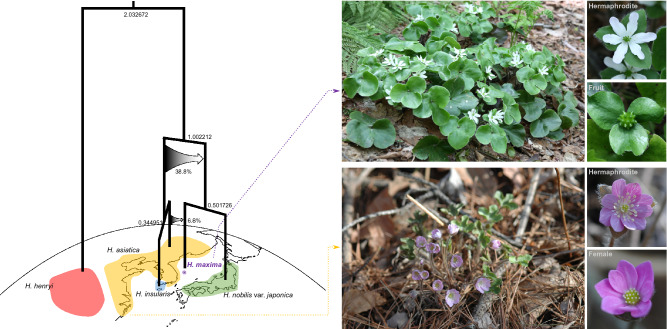
Summary of two introgression events associated with the origin of *H. maxima* on chronogram. Numbers at nodes indicated divergence time estimate in Mya. See supplementary Fig. S5 for more detail for tribe Anemoneae. Arrows indicate introgressions with the inferred inheritance probability. Color areas indicate the geographic distribution of each species. The images were constructed in InkScape v0.92.2 (https://inkscape.org) based on the chronogram. The top right corner of the photos shows habit, flower, and fruits (mature green) of *H. maxima*, and the bottom right corner of the photos shows the habit and hermaphroditic and female flowers of *H. asiatica*.

We further used the 526-gene dataset, excluding the two outgroups, to infer reticulate evolution in *Hepatica* by PhyloNet. PhyloNet analyses revealed a complex network of ancestral hybridization among the Asian lineages of *Hepatica*, with five reticulations selected by the Akaike information criterion as the best fit (Fig. 6 and Supplementary Table S9). The PhyloNet network with the highest likelihood (-1030.49400400281) indicated that ancient hybridization and introgression events occurred within four species, thus revealing a complicated evolutionary history of *H. asiatica* that involved hybridization and introgression among extinct species (Fig. 6). The gene flow events and divergence time estimates for *H. maxima* are summarized in Fig. 7. Our results indicated that the most recent common ancestor of crown *Hepatica* appeared ~ 2.03 Mya (95% highest posterior density (HPD) = 1.07–3.08, Supplementary Fig. S5). One of the lineages split into two new lineages 1.00 Mya (95% HPD = 0.64–1.45, Supplementary Fig. S5): (1) the ancestral lineage of *H*. *maxima*/*H*. *nobilis* var. *japonica* and the ancestral lineage of *H*. *insularis*/*H*. *asiatica*. The inheritance probability showed that the ancestral lineage of *H*. *maxima*/*H*. *nobilis* var. *japonica* had a genomic contribution of 38.8% from the ancestral lineage of *H*. *insularis*/*H*. *asiatica* between 0.34 and 1 Mya and that a minority of the genetic material composing the *H. maxima* lineage (6.8%) was derived from the ancestral branch of *H. asiatica*, which occurred < 0.34 Mya (Fig. 7).

## Discussion

Oceanic islands provide good model systems for studying divergence and plant speciation because they are geologically young and isolated and have diverse habitats^1^. Many island plants have diverse and complex evolutionary histories involving various processes, such as rapid adaptation, hybridization, and introgression^31–33^. Genome-scale data provide a powerful source for studying evolution in island plants^34^. In this study, we first generated the complete plastid and mitochondrial genomes of *H. maxima*, one of the endemic plants observed on the oceanic Ulleung island, Korea, which is known to have originated via anagenetic speciation. Compared with the ancestral angiosperm genome, the *H. maxima* plastome exhibits structural divergence, although its organization is identical to that previously described in *Hepatica* species^35^. Additional genome sequences from *Hepatica* revealed that plastomes of the *Hepatica* lineage have low rates of sequence variation and structural evolution. A comparison of the *Hepatica* plastomes with those of four representative genera from tribe Anemoneae provides insight into structural evolutionary events, including inversions and gene losses (Fig. 3). Plastome rearrangements in tribe Anemoneae have been documented^35,36^. Our results are consistent with the previous inference of six inversion events^36^, although the order of inversion events is slightly different. Our results suggested that the loss of *trnT-UGU* may be associated with one of these inversion events. The losses of the plastid *infA* and *rpl32* genes in tribe Anemoneae were revealed as the result of ongoing functional transfer into the nucleus. The finding of the nuclear-encoded plastid *rps16* gene was found in all additional transcriptomes, suggesting that functional replacement by gene substitution occurred in the common ancestor of tribe Anemoneae (Fig. 3). The coexistence of plastid and nuclear *rps16* homologs within tribe Anemoneae (except in *Hepatica*) indicates that functional transfer to the nucleus is necessary before the loss of the original plastid copy.

In angiosperms, the mitogenomes of approximately one hundred genera have been sequenced, which only represents 0.77% of 13,164 genera^37^, thus increasing the difficulty of performing comparative analyses within species, genera, and families. The *H. maxima* mitogenome represents the first complete mitogenome from the order Ranunculales and shows a relatively large size compared to that of the *Nelumbo nucifera* mitogenome (524,797 bp, NC_030753) from Proteales and exhibits the loss of two mitochondrial genes. We also found evidence of mitochondrion-to-nucleus transfer of *rps10* and *rps14* in *H. maxima* (Fig. 4). The *H. maxima* mitogenome contains eighteen chimeric ORFs, five of which have partial mitochondrial genes and transmembrane helices. In particular, *orf297* and *orf296* include a portion of *atp1*, suggesting that these ORFs have the potential to cause cytoplasmic male sterility (CMS)^38^. The chimeric ORFs with an ATP synthase subunit are frequently associated with genome rearrangements^38,39^. In particular, *orf296* overlapped with one copy of repeats that are collapsed to call the other contigs (Supplementary Fig. S2), which may indicate that the repeats are associated with alternative arrangements resulting from homologous recombination. The chimeric structure of a partial *atp1* and association with the repeat are similar to that of known CMS-associated ORFs in *Mimulus*^39^. Further work is needed to determine if these ORFs are expressed and functional. The finding of a candidate CMS gene in the *H. maxima* mitogenome is of particular interest due to the presence of gynodioecy within the genus, which has been documented in *H. nobilis*^40^. We also observed both hermaphroditic and female individuals within a natural population of *H. asiatica* (Fig. 7) in southern South Korea. The candidate CMS gene may be useful for studies of gynodioecious populations of *H. asiatica.*

Large-scale genomic data enabled us to understand the evolutionary history of Asian *Hepatica,* including the insular endemic species *H. maxima*. Our results showed clear evidence of cytonuclear and organellar discordance (Fig. 5), indicating that Asian *Hepatica* may have experienced complicated evolutionary events. For example, the conflict between two organellar genomes suggests differential inheritance of organelles in the Asian *Hepatica* lineages, which resulted in the disruption of linkage disequilibrium (LD) between plastids and mitochondria^41^. The presence of CMS in gynodioecious systems can select for the paternal leakage of their mitochondrial genomes^21^. In Asian *Hepatica*, a gynodioecious population and potential CMS genes were observed, supporting the possibility of mitochondrial heteroplasmy in the lineages. Consequently, patterns of cytonuclear discordance in the Asian *Hepatica* lineages were also identified (Fig. 5). Cytonuclear discordance is known to be caused by hybridization and introgression, and these phenomena are common in plants because of organellar genome capture^42^. Taken together, the phylogenomic incongruence and the potential CMS implied that Asian *Hepatica* has experienced reticulation events (such as hybridization, introgression and horizontal gene transfer) or stochastic processes, including incomplete lineage sorting (ILS), via the biparental inheritance of organelles, although the incongruence is likely caused by insufficient parsimony-informative sites from the plastid and mitochondrial genes. The rapid diversification and gene tree discordance also indicated reticulate evolution in the Asian *Hepatica* lineage.

Our PhyloNet results provide strong evidence that hybridization and introgression events have contributed to the evolutionary history of Asian *Hepatica* (Fig. 6). In particular, *H. asiatica* has an extremely complex history (Fig. 6). The species network revealed that the evolution of the insular endemic species *H. maxima* involved two events: (1) massive ancient introgression (38.8%) from the common ancestor of *H. asiatica*/*H. insularis* to the common ancestor of *H. maxima*/*H. nobilis* var. *japonica* and (2) recent introgression (6.8%) from *H. asiatica* to *H. maxima* (Fig. 7). Recent introgression suggested that the ancestral population of *H. maxima* would have been very morphologically similar to the ancestor of *H. asiatica* and that interbreeding was possible. Genetic changes due to ancient and recent introgression events may be an important source for anagenetic speciation on Ulleungdo. During anagenetic speciation, *H. maxima* developed increased leaf, bract and seed sizes compared to those of the putative progenitor, which is a process known as “insular gigantism”. Functional analyses of polymorphisms in the *H. maxima* transcriptome associated with cell division (e.g., leaf, bract and seed sizes) require further study to provide valuable insights into adaptation to islands. A previous study suggested that *H. maxima* was derived from the Korean mainland species *H. asiatica*^12^. However, our results suggested that the insular endemic species *H. maxima* originated from an ancestral lineage that split from the common ancestor of *H. maxima*/*H. nobilis* var. *japonica*.

## Materials and methods

### DNA and RNA sequencing

Hermaphroditic *Hepatica maxima* individuals were collected from Ulleungdo, Korea. We prepared total genomic DNA (gDNA) from fresh leaf tissue using a DNeasy Plant Mini Kit (Qiagen, Hilden, Germany). The DNA was sequenced from one PE read from a 550 bp library and two mate pair (MP) reads from 3000 and 8000 bp libraries using the Illumina HiSeq 2000 sequencing platform (Illumina, San Diego, CA). Approximately 16 Gb of PE, 17 Gb of 3 kp MP, and 10 Gb of 8 kp MP reads were generated. Additional gDNA was extracted using a Bead Genomic DNA Prep Kit For Plants (A_type, BIOFACT Co., Daejeon, South Korea), and long reads were sequenced using the ONT GridlON platform (ONT, Oxford, United Kingdom). Total RNA was isolated as described by Zhang et al.^43^ and sequenced using the Illumina HiSeq 2000 sequencing platform.

Additional gDNA and RNA extractions from four *Hepatica* species (*H. asiatica*, *H. henryi*, *H. insularis*, and *H. nobilis* var. *japonica*) and the two outgroups *A. narcissiflora* and *P. koreana* were performed as described above. The six sets of gDNA and RNA were sequenced in only the PE library using the Illumina platform.

### Organellar genome assembly and annotation

To complete the seven plastomes, the PE reads from gDNA were assembled de novo with Velvet v1.2.10^44^ using multiple *k*-mers (69 to 95). For the *H. maxima* mitogenome, we performed hybrid assemblies with the PE/MP and Nanopore reads using Spades v3.13.1^45^ and MaSuRCA v3.2.6^46^. The contigs from the multiple assemblies were aligned manually in Geneious R7 v7.1.8 (www.geneious.com)^47^, and the mitogenome was completed by tracking and end inspecting the contigs. To assess the depth of coverage, the Illumina and Nanopore reads were mapped to the genomes with Bowtie2 v2.2.9^48^ and Minimap2 v2.17^49^, respectively. Plastome annotation was performed as described previously^50^, and the mitogenome was annotated using Mitofy^51^. Repeats were identified by performing BLAST + v2.10.0^52^ comparisons of the *H. maxima* mitogenome against itself with an e-value cutoff of 1 × 10^–6^ and a word size of 16. Plastid-derived sequences in the mitogenome were identified by performing “blastn” searches of the *H. maxima* plastome against the mitochondrial genome with an e-value cutoff of 1 × 10^–6^, a sequence identity of at least 80% and a minimum length of 50 bp. RNA editing sites were predicted using PREP-Mt^53^ with a cutoff value of 0.5. To identify chimeric ORFs in the *H. maxima* mitogenome, all ORFs at least 150 bp in length were extracted from intergenic regions. We searched for ORFs containing one or more mitochondrial gene fragments using “blastn” with an e-value cutoff of 1 × 10^–3^, a minimum length of 30 bp, and a sequence identity of at least 90%. Transmembrane domains in the identified chimeric ORFs were predicted using TMHMM v2.0^54^. The *H. maxima* organellar genome maps were drawn with OGDRAW v1.3.1^55^, and the genome sequences were deposited in GenBank under accession numbers MG952899 and MT568500.

For the other species, mitochondrial genes were identified in each draft assembly by a blastn search using the protein-coding genes from the *H. maxima* mitogenome as query sequences.

### Nuclear transcriptome assembly and orthology inference

Five *Hepatica* and two outgroup de novo transcriptome assemblies were performed using Trinity v2.5.1^56^ with default parameters. We then used TransRate^57^ to filter “bad transcripts” that included chimeras, structural errors, incomplete assemblies, and base errors. To greatly reduce the number of redundant transcripts, highly similar copies among the filtered transcripts were clustered using CD-HIT v4.8.1^58^ with an amino acid identity threshold of 0.95 (cd-hit-est). Finally, the clustered transcripts were filtered to obtain the longest isoform per Trinity gene. TransDecoder v5.5.0 (https://transdecoder.github.io) was then used to identify the best ORF for each transcript. Single-copy orthologs were inferred with OrthoFinder2 v2.3.11^59^ using the multiple sequence alignment (MSA) program with MAFFT v7.271^60^ and IQ-TREE v2.0^61^.

### Detection of functional replacement events

IGT events were identified using “blastn” (e-value cutoff of 1 × 10^–6^) searches of the plastid-encoded genes (*infA* and *rpl32* from *Megaleranthis saniculifolia*; NC_012615), mitochondrial-encoded genes (*rps10* and *rps14* from *Nicotiana tabacum*; NC_006581), and nuclear-encoded genes (*rps16* from *Medicago truncatula*; AB365526) against the five *Hepatica* and two outgroup transcriptomes. TargetP v.1.1^62^ was used to predict plastid or mitochondrial transit peptides. The NCBI Conserved Domain Database (CDD) v3.18^63^ was used for functional domain annotation. Nucleotide and amino acid sequences of nuclear and mitochondrial genes were aligned with MUSCLE^64^ in Geneious R7.

### Phylogenomics and divergence time analyses

Seventy-six plastid-encoded and thirty-seven mitochondrial-encoded genes were shared by the seven species (Supplementary Table S5). For nuclear genes, we excluded single-copy orthologs with possible sequence biases and misleading signals due to long-branch attraction and saturation using TreSpEx v1.1^65,66^. The individual gene alignments were generated based on the back-translation approach with MAFFT.

Phylogenomic analyses were performed with concatenation‐ and coalescent‐based methods. For the concatenation method, nucleotide sequences of 526, 413, 76 and 37 genes of the five *Hepatica* species and two outgroups were concatenated into four large matrices. ML trees were constructed by IQ-TREE under the best-fit substitution model from each of the 526, 413, 76 and 37 partitioning schemes with the ultrafast bootstrap algorithm (1,000 replicates). The gene and site concordance factors (gCF and sCF, respectively) were also computed by IQ-TREE. For the coalescent‐based method, single‐gene trees were constructed using IQ-TREE under the best substitution model by ModelFinder, and then the program ASTRAL v5.7.1^67^ was used for the datasets of 526, 413, 76 and 37 genes. The split network was inferred with SplitsTree v4.15.1^68^ using the Uncorrected_P and NeighborNet methods and 1000 bootstrap replicates. The Φ_W_ (PHI) test^69^ for detecting recombination was computed using SplitsTree. Recombination detected by Gubbins^70^ was removed, and the resulting alignments were then analyzed by IQ-TREE to generate a ML phylogeny.

To reconstruct and evaluate the reticulate evolutionary relationships among the five *Hepatica* species, species networks that modeled ILS and introgression using a ML approach were constructed in PhyloNet v3.8.2^71^ with the command “InferNetwork_ML” and using the individual ML gene trees. Network searches were performed using only nodes in the rooted ML gene trees that had a bootstrap support of at least 75%, which allowed for zero to six reticulations, and with optimization of the branch lengths and inheritance probabilities of the returned species networks under pseudo-likelihood.

We estimated the divergence times by using Bayesian Markov chain Monte Carlo (MCMC) methods in BEAST2 v2.6.2^72^ with the concatenated alignment dataset. A calibration point estimate for tribe Anemoneae^36^ (mean = 22.5, SD = 5.35, range = 13.7–31.3) was used with a normal prior distribution as the root age constraint.

## Supplementary information

Supplementary Information.

## Data Availability

Complete plastid and mitochondrial genomes and nuclear-encoded gene sequences have been submitted to GenBank (accession numbers MG952899, MT568500, MT560532-MT560561). The phylogenomic datasets generated during the current study are available in the Dryad Digital Repository, [https://doi.org/10.5061/dryad.s1rn8pk50].
