# Supplementary material for: Large-scale phylogenomics reveals ancient introgression in Asian Hepatica and new insights into the origin of the insular endemic Hepatica maxima
Source: Sci Rep. 2020 Oct 1;10:16288. doi: 10.1038/s41598-020-73397-2 (PMC7529770; doi:10.1038/s41598-020-73397-2)
Supplement: Supplementary file 1 — Supplementary Information. [file 41598_2020_73397_MOESM1_ESM.pdf]

Article title: Large-scale phylogenomics reveals ancient introgression in Asian *Hepatica* and new insights into the origin of the insular endemic *Hepatica maxima*

Authors: Seongjun Park and SeonJoo Park

Supplementary information

Figure S1. Prediction of transmembrane helices in ORFs that contain a mitochondrial gene.

Figure S2. Mitochondrial genomic map surrounding the *orf296*.

Figure S3. Structural alignments of tribe Anemonea plastomes using Mauve.

Figure S4. Phylogenetic relationships among 5 species of the Asian *Hepatica* with two outgroups.

Figure S5. Chronogram of the Asian *Hepatica* divergence times.

Table S1. Blast result of plastid-derived DNA segments in the mitochondrial genome of *Hepatica maxima*.

Table S2. Predicted RNA editing in 39 protein-coding genes for the *Hepatica maxima* mitochondrial genome.

Table S3. Potential chimeric ORFs.

Table S4. General characteristics of newly sequenced plastomes.

Table S5. Organellar genes included in phylogenomic analyses.

Table S6. Transit peptide prediction scores of putative nuclear-encoded plastid and mitochondrial genes with GenBank accession numbers.

Table S7. General characteristics of nuclear orthologs .

Table S8. Scores of each criterion calculated by TreSpEx.

Table S9. Model selection between species networks.

Figure S1. Prediction of transmembrane helices in ORFs that contain a mitochondrial gene.

Transmembrane helices are indicated by vertical red lines. The inside and outside represents the orientation of helices relative to the mitochondrial membrane

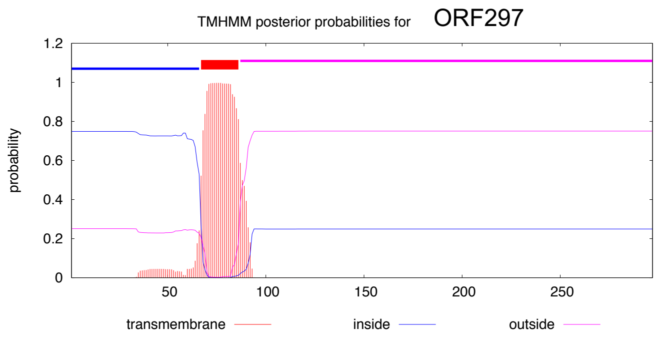

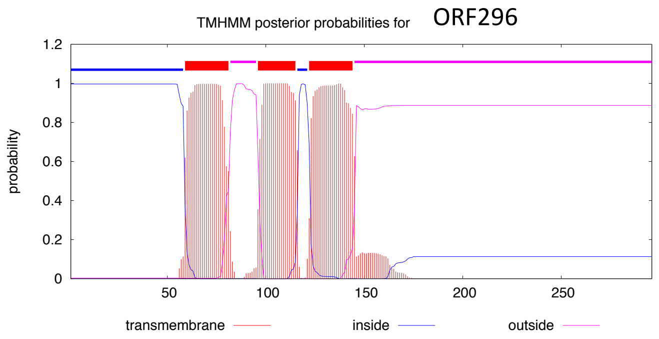

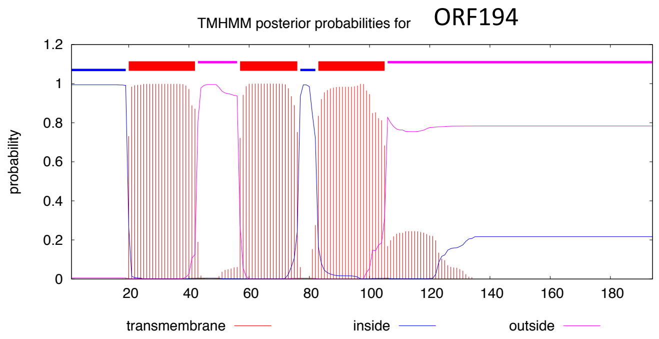

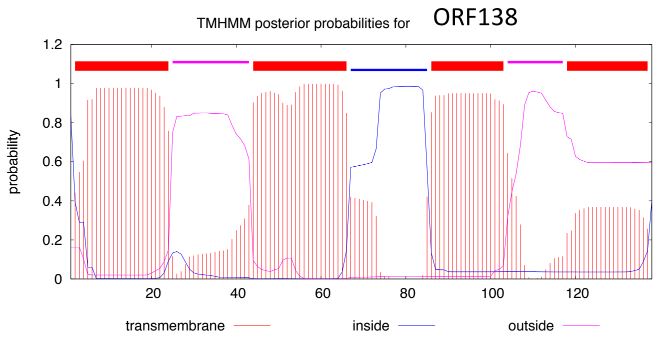

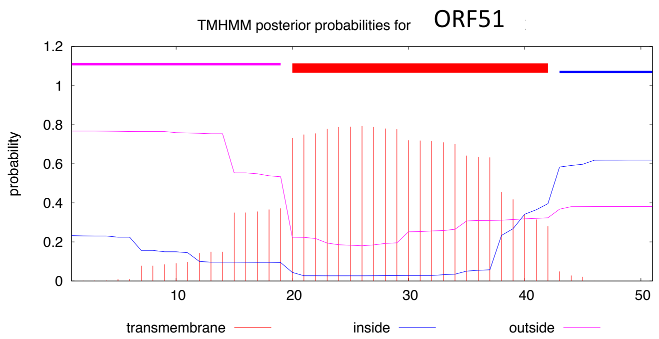

Figure S2. Mitochondrial genomic map surrounding the *orf296*.

The three repeats are shown in black. The repeats are shown in all nine possible arrangements. Transmembrane helices are indicated by vertical red lines. The inside and outside represents the orientation of helices relative to the mitochondrial membrane (see Supplementary Fig. S1 for more detail). The figures were constructed in OGDRAW v1.3.1 (https://chlorobox.mpimp-golm.mpg.de/OGDraw.html) and InkScape v0.92.2 (https://inkscape.org).

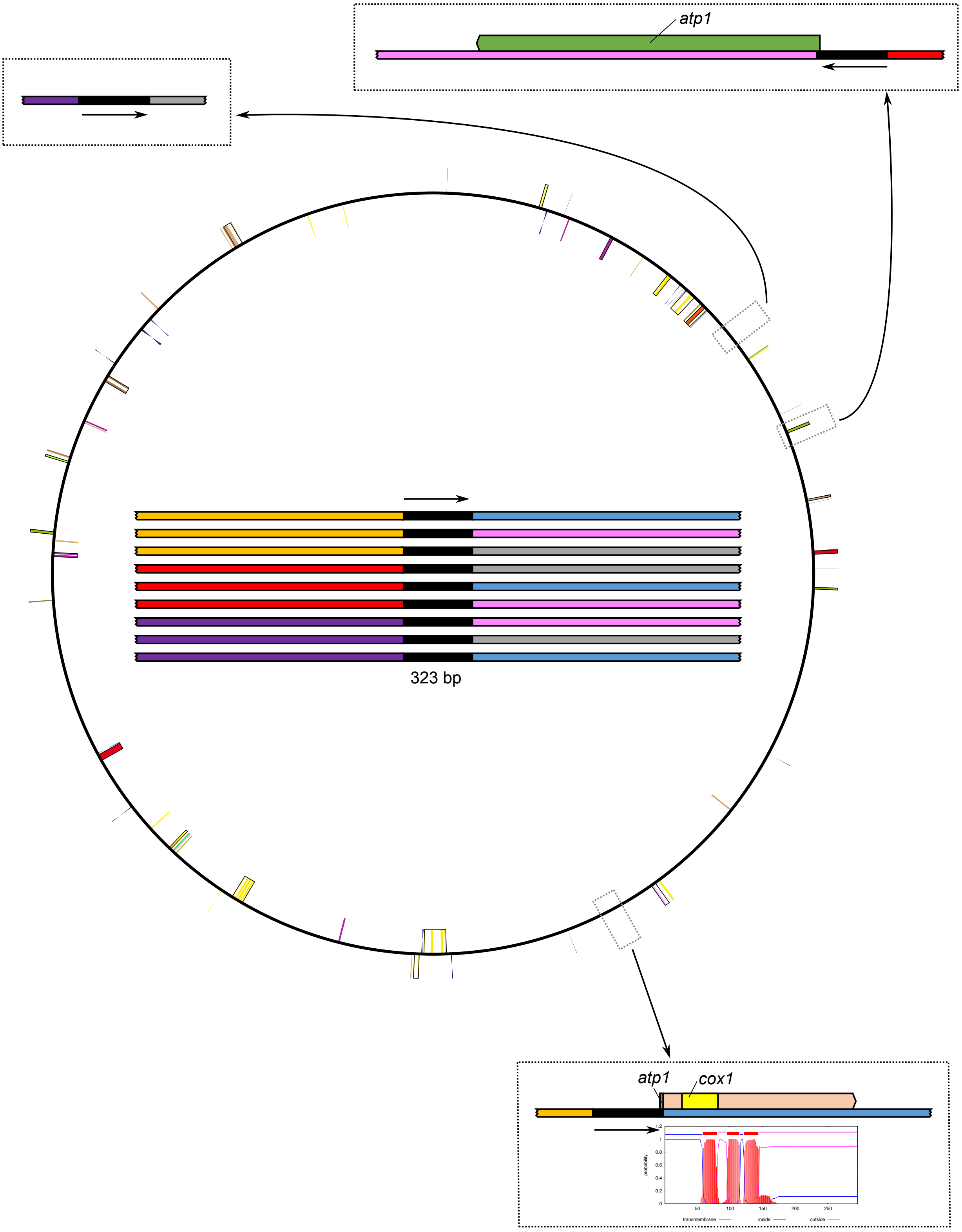

Figure S3. Structural alignments of tribe Anemonea plastomes using Mauve.

The colored blocks represent collinear sequence blocks shared by all plastomes. Blocks drawn below the horizontal line indicate sequences found in an inverted orientation. Individual genes and strandedness are represented below the *Ranunculus* genome block. Only one copy of the inverted repeat (IR) is shown for each plastome and pink boxes below each plastome block indicate its IR. The figures were constructed using Mauve v2.3.1 (http://darlinglab.org/mauve/mauve.html) in Geneious R7 v7.1.8 (https://www.geneious.com).

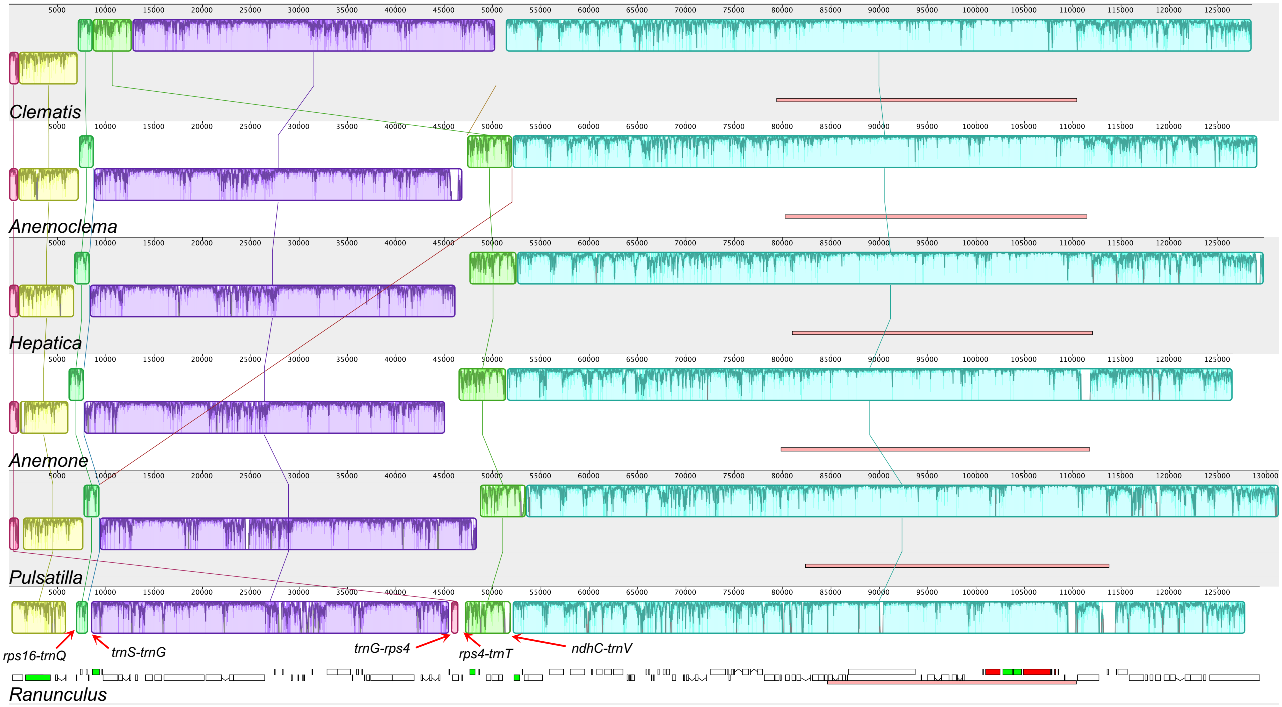

Figure S4. Phylogenetic relationships among 5 species of the Asian *Hepatica* with two outgroups. The maximum likelihood tree was constructed using nucleotide sequence of 413 nuclear (A), 76 plastid (B) and 37 mitochondrial (C) genes, respectively. Bootstrap support values > 50% are shown at nodes. The figures were constructed in IQ-TREE v2.0 (http://www.iqtree.org/).

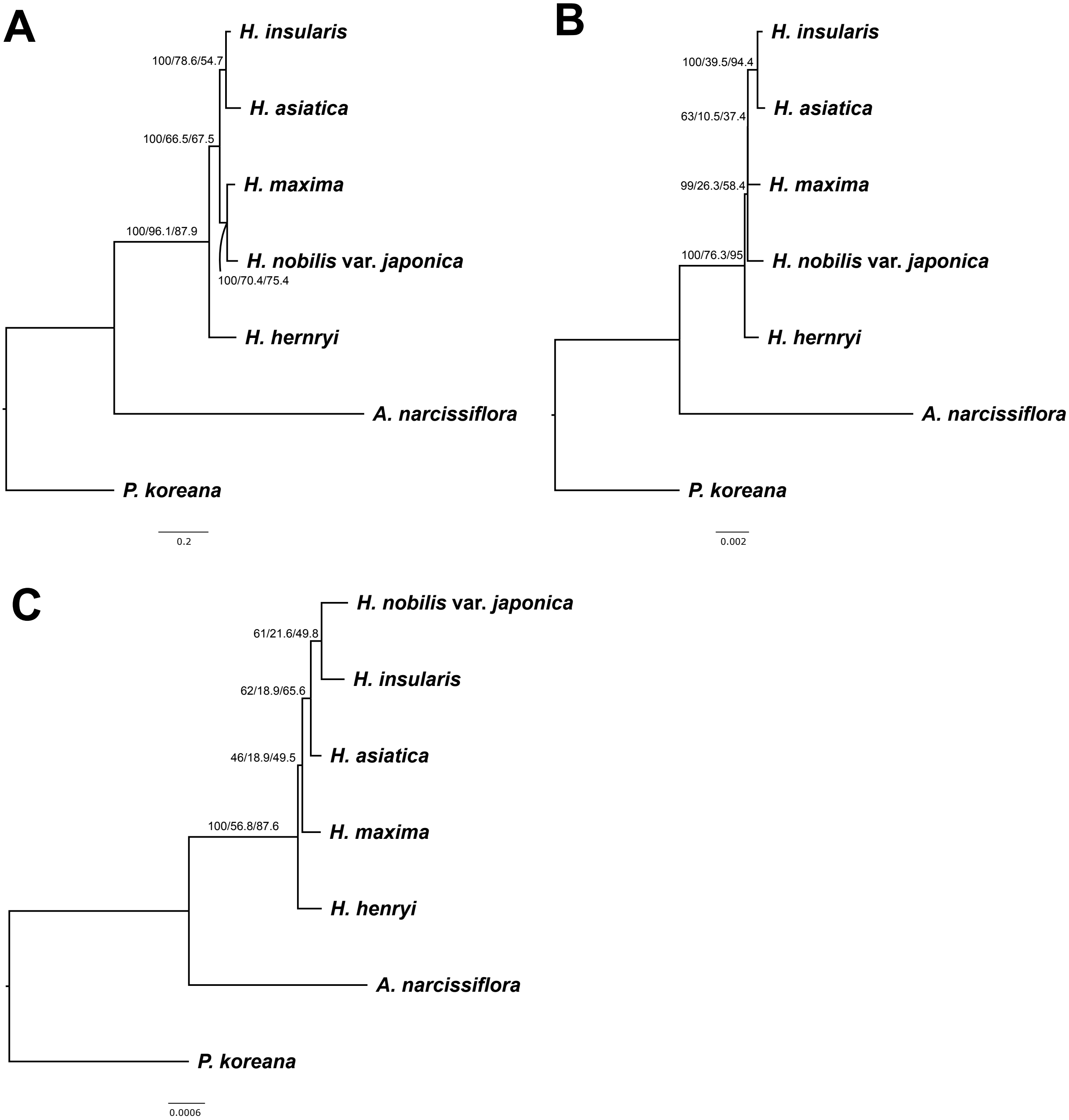

Figure S5. Chronogram of the Asian *Hepatica* divergence times. Times shown are the median age estimates from the BEAST analysis (blue bars indicate 95% highest posterior density [HPD] intervals). The figures were constructed in BEAST2 v2.6.2 (https://www.beast2.org/).

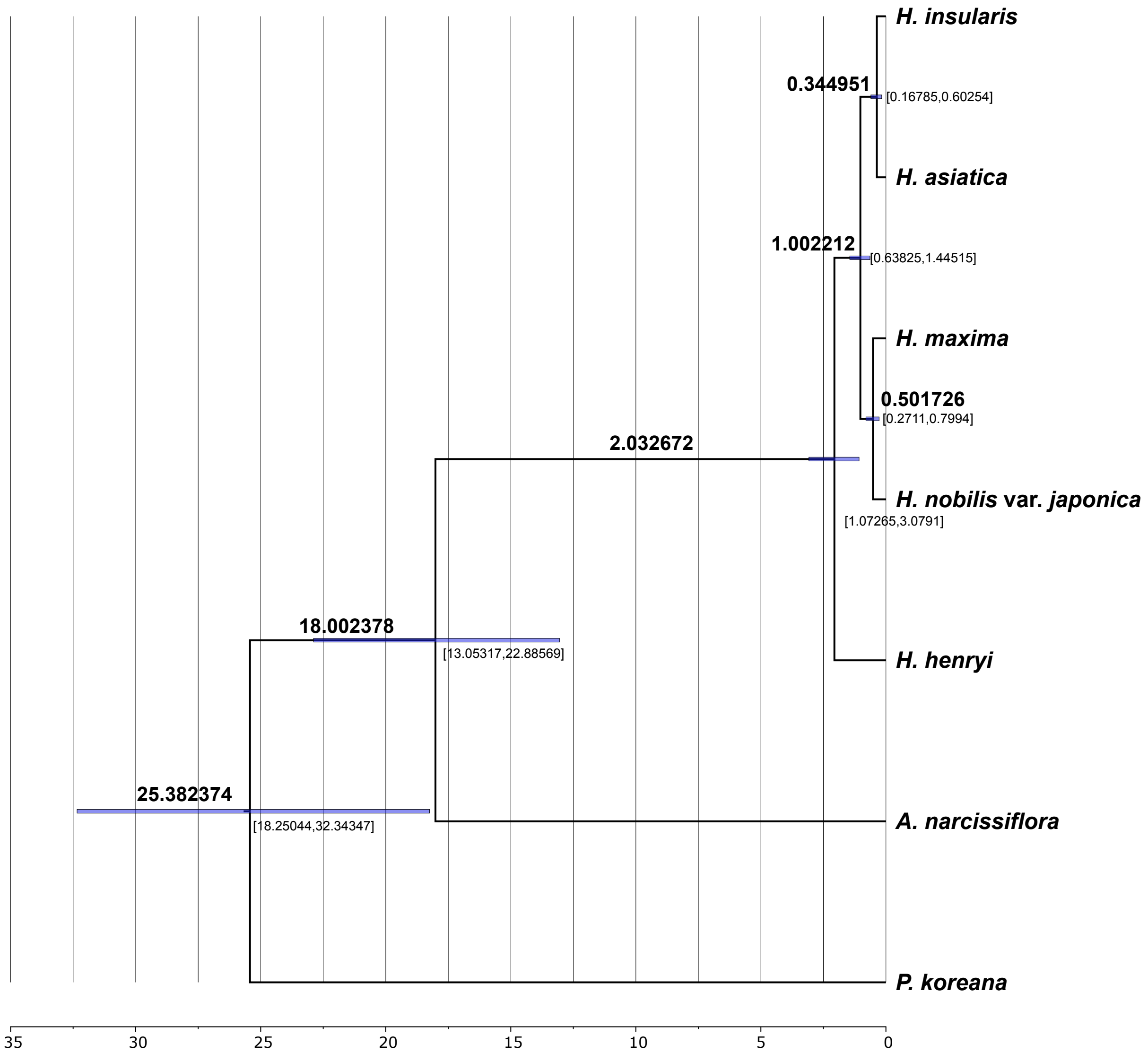

Supplementary Table S1. Blast result of plastid-derived DNA segments in the mitochondrial genome of *Hepatica maxima*. Asterisks indicate fragments.

	Mt-length	Pt-length	Identigy	Aligned length	Query start	Query end	Hit start	Hit end	E value	Bit-score	Annotated plastid genes
1	7104	7103	99.339	7108	12340	19442	107497	114600	0	12857	psaA*, psaB, rps14, trnfM, trnG, psbZ, trnS, psbC*
2	5680	5680	99.982	5680	132849	138528	963119	957440	0	10484	trnI, trnA, 23S rRNA, 4.5S rRNA, 5S rRNA
3	895	952	80.992	968	67127	68078	907075	906181	0	686	trnP, trnW, petG, petL
4	884	884	99.548	884	101755	102638	583440	582557	0	1611	16S rRNA*
5	643	651	97.081	651	19631	20281	883235	882593	0	1090	psbC*, psbD*
6	214	208	87.383	214	10474	10681	356013	355800	4.50E-62	241	ycf3 intron2*
7	166	161	90.964	166	23782	23942	850512	850677	2.11E-55	219	trnD
8	148	158	83.019	159	28848	29005	871312	871459	7.85E-30	134	rpoB*
9	104	104	89.423	104	154421	154524	274099	273996	2.82E-29	132	
10	102	102	94.118	102	88025	88126	810091	809990	1.68E-36	156	ycf2*
11	86	75	84.884	86	87501	87575	274266	274181	1.34E-12	76.8	trnI
12	84	83	95.238	84	131821	131903	152020	151937	2.82E-29	132	trnN
13	82	80	80.488	82	59598	59677	872114	872195	3.76E-08	62.1	accD*
14	80	80	97.5	80	6886	6965	836794	836715	6.07E-31	137	trnH(GUG)
15	76	78	92.308	78	6887	6964	436560	436485	1.32E-22	110	trnH(GUG)
16	72	72	82.667	75	117893	117964	31025	30954	3.76E-08	62.1	ycf1*
17	75	75	86.667	75	151327	151401	692214	692140	8.02E-15	84.2	ycf2*

18	75	72	92	75	22382	22453	850260	850334	2.21E-20	102	trnT
19	75	75	93.333	75	53734	53808	1034272	1034346	3.68E-23	111	trnM
20	73	73	93.151	73	75138	75210	67018	66946	4.76E-22	108	psbB*
21	73	73	93.151	73	75138	75210	114840	114912	4.76E-22	108	psbB*
22	73	73	93.151	73	75138	75210	929634	929562	4.76E-22	108	psbB*
23	69	69	82.857	70	105869	105937	653600	653532	3.76E-08	62.1	23S rRNA*
25	61	61	86.885	61	140008	140068	8934	8874	2.24E-10	69.4	16S rRNA*
26	61	61	86.885	61	101807	101867	176307	176247	2.24E-10	69.4	16S rRNA*
27	54	54	98.148	54	96538	96591	101217	101164	3.70E-18	95.3	ndhB intron*
28	54	54	100	54	101446	101499	1119593	1119540	7.96E-20	100	
29	51	51	88.235	51	138596	138646	10526	10476	3.76E-08	62.1	16S rRNA*
30	51	50	90.196	51	32260	32309	230294	230344	2.90E-09	65.8	rpoC1 intron*/nad5 intron

Supplementary Table S2. Predicted RNA editing in 39 protein-coding genes for the *Hepatica maxima* mitochondrial genome.

Sequence Name	Length	RNA editing	Sequence Name	Length	RNA editing
atp1	1,548	3	*nad5*	2,013	36
atp4	564	14	*nad6*	825	17
atp6	1,140	25	*nad7*	1,185	38
atp8	486	5	*nad9*	573	12
atp9	225	1	*rpl2*	1,536	5
ccmB	621	40	*rpl5*	558	9
ccmC	744	37	*rpl10*	489	5
ccmFc	1,356	23	*rpl16*	516	9
ccmFn	1,734	40	*rps1*	690	3
cob	1,182	22	*rps2*	711	7
cox1	1,587	28	*rps3*	1,695	14
cox2	765	15	*rps4*	1,188	20
cox3	798	19	*rps7*	447	2
matR	1,971	20	*rps11*	771	4
mttB	815	34	*rps12*	378	10
nad1	787	33	*rps13*	351	6
nad2	1,467	35	*rps19*	288	3
nad3	357	18	*sdh3*	336	2
nad4	1,488	57	*sdh4*	567	5
nad4L	273	12			

Supplementary Table S3. Potential chimeric ORFs.

		ORF start	ORF end	ORF length	Identity	ORF hit start	ORF hit end	Chimera length	Gene hit start	Gene hit end	E-value	Gene	No of Transmembrane helices / probabilities
1	**ORF297**	**11083**	**11976**	**894**	**96.825**	**4**	**66**	**63**	**1**	**63**	**6.07E-24**	***atp1***	**1 / 0.74887**
2	**ORF296**	**929844**	**930732**	**891**	**96.875**	**4**	**35**	**32**	**1**	**32**	**2.23E-08**	***atp1***	**3 / 0.99653**
					**91.515**	**118**	**282**	**165**	**1**	**162**	**1.65E-59**	***cox1***	
3	**ORF194**	**462818**	**462234**	**585**	**92.727**	**1**	**162**	**165**	**1**	**165**	**7.61E-63**	***cox1***	**3 / 0.99422**
4	**ORF138**	**772394**	**771978**	**417**	**96.324**	**1**	**136**	**136**	**1**	**136**	**2.08E-59**	***nad6***	**4 / 0.83666**
5	ORF129	353926	354315	390	98.75	188	110	80	203	282	2.97E-35	*rps19*	0 / 0.02742
6	ORF126	771025	771405	381	98.438	278	151	128	615	740	1.40E-59	*ccmFc*	0 / 0.07420
7	ORF112	12267	11929	339	100	43	1	43	50	92	1.94E-16	*nad6*	0 / 0.18937
					98.925	249	157	93	549	641	2.81E-42	*cox1*	
9	ORF102	543488	543180	309	95.161	248	309	62	25	86	1.39E-22	*atp9*	0 / 0.26823
10	ORF98	431052	431348	297	90.6	71	102	32	155	186	4.26E-07	*rrn26*	0 / 0.12362
11	ORF90	324169	323897	273	100	1	251	251	205	455	8.91E-132	*cox1*	0 / 0.23289
12	ORF77	41289	41522	234	93.333	46	17	30	3087	3116	2.97E-05	*rrn26*	0 / 0.10158
13	ORF63	802201	802392	192	96.97	188	156	33	510	542	4.54E-09	*atp6*	0 / 0.76896
14	ORF59	1034878	135057	180	100	46	162	117	1505	1621	5.94E-57	*rrn26*	0 / 0.27770
15	ORF56	424903	424733	171	90.244	117	37	82	23	104	8.30E-25	*atp9*	0 / 0.03328
16	ORF53	695973	696134	162	97.297	39	75	37	37	1	1.95E-11	*nad6*	0 / 0.61660
	ORF52	1034984	1034826	159	100	62	1	62	1505	1566	2.23E-26	*rrn26*	0 / 0.12545
17	**ORF51**	**419736**	**419891**	**156**	**97.778**	**133**	**89**	**45**	**916**	**960**	**1.01E-15**	***rps4***	**1 / 0.23233**
18	ORF50	223906	224058	153	98.113	46	98	53	945	997	3.61E-20	*rps4*	0 / 0.57962

Supplementary Table S4. General characteristics of newly sequenced plastomes.

Taxon	Plastome size (bp)	LSC length (bp)	SSC length (bp)	IR length (bp)	Protein-coding genes	tRNA genes	rRNA genes	GC content (%)	GenBank accession number
***Hepatica* Mill.**									
H. asiatica	160,157	80,343	17,778	31,018	76	29	4	37.9	MG952902
H. henryi	159,892	80,779	17,029	31,042	76	29	4	37.9	MG952901
H. insularis	160,423	80,538	17,831	31,027	76	29	4	37.8	MG952900
H. maxima	160,876	80,998	17,684	31,097	76	29	4	37.8	MG952899
H. nobilis var. *japonica*	160,988	80,996	17,792	31,100	76	29	4	37.8	MG952898
**Outgroup**									
Anemone narcissiflora	158,557	79,829	14,784	31,972	77	29	4	37.7	MG952903
Pulsatilla koreana	162,709	82,355	17,544	31,405	77	29	4	37.4	MG952897

Supplementary Table S5. Organellar genes included in phylogenomic analyses.

Gene Groups (abbreviation)	Genes
**Plastid**	
ATP synthase (*atp*)	*atpA, atpB, atpE, atpF, atpH, atpI*
Cytochrome b6/f (*pet*)	*petA, petB, petD, petG, petL, petN*
NADH-plastoquinone oxidoreductase (*ndh*)	*ndhA, ndhB, ndhC, ndhD, ndhE, ndhF, ndhG, ndhH, ndhI, ndhJ, ndhK*
Photosystem I (*psa*)	*psaA, psaB, psaC, psaI, psaJ*
Photosystem II (*psb*)	*psbA, psbB, psbC, psbD, psbE, psbF, psbH, psbI, psbJ, psbK, psbL, psbM, psbN, psbT, psbZ*
Ribosomal protein, large subunits (*rpl*)	*rpl2, rpl14, rpl16, rpl22, rpl33, rpl36*
Ribosomal protein, small subunits (*rps*)	*rps2, rps3, rps4, rps7, rps8, rps11, rps12, rps14, rps15, rps19*
RNA polymerase (*rpo*)	*rpoA, rpoB, rpoC1, rpoC2*
Others	*ccsA, cemA, clpP, rbcL, ycf1, ycf2, ycf3, ycf4, matK*
**Mitochondrion**	
ATP synthase (*atp*)	*atp1, atp4, atp6, apt8, atp9*
Cytochrome c Maturation (*ccm*)	*ccmB, ccmC, ccmFc, ccmFn*
Cytochrome bc1 complex	*cob*
Cytochrome c oxidase (*cox*)	*cox1, cox2, cox3*
NADH-ubiquinone oxidoreductase (*nad*)	*nad1, nad2, nad3, nad4, nad4L, nad5, nad6, nad7, nad9*
Ribosomal proteins, large subunit	*rpl2, rpl5, rpl10, rpl16*
Ribosomal proteins, small subunit	*rps2, rps3, rps4, rps7, rps11, rps12, rps13*
Others	*matR, mttB, sdh4*

Supplementary Table S6. Transit peptide prediction scores of putative nuclear-encoded plastid and mitochondrial genes with GenBank accession numbers. cTP = chloroplast transit peptide. mTP = a mitochondrial targeting peptide. RC indicates reliability class, from 1 to 5, where 1 indicates the strongest prediction. Tplen means predicted presequence length (cleavage sites). Bold font and Loc indicate prediction of localization (chloroplast or mitochondrion).

Gene	Taxa	Len	cTP	mTP	SP	other	Loc	RC	TPlen	GenBank
nuclear-encoded *infA* for plastids	*H. asiatica*	149	**0.940**	0.065	0.012	0.058	C	1	62	MT560533
	H. henryi	148	**0.905**	0.044	0.027	0.080	C	1	61	MT560536
	H. insularis	149	**0.940**	0.065	0.012	0.058	C	1	62	MT560534
	H. maxima	149	**0.929**	0.072	0.013	0.049	C	1	62	MT560535
	H. nobilis var. *japonica*	149	**0.930**	0.070	0.013	0.050	C	1	62	MT560532
	Anemone narcissiflora	148	**0.905**	0.041	0.029	0.106	C	2	61	MT560537
	Pulsatilla koreana	138	**0.840**	0.158	0.035	0.070	C	2	58	MT560538
nuclear-encoded *rpl32* for plastids	*H. asiatica*	177	**0.845**	0.375	0.004	0.016	C	3	55	MT560540
	H. henryi	177	**0.895**	0.189	0.008	0.038	C	2	85	MT560544
	H. insularis	179	**0.858**	0.286	0.005	0.021	C	3	26	MT560541
	H. maxima	177	**0.862**	0.315	0.004	0.023	C	3	85	MT560542
	H. nobilis var. *japonica*	177	**0.862**	0.315	0.004	0.023	C	3	85	MT560543
	Anemone narcissiflora	163	**0.692**	0.548	0.008	0.023	C	5	40	MT560539
	Pulsatilla koreana	154	**0.915**	0.238	0.004	0.021	C	2	71	MT560545
nuclear-encoded *rps10* for mitochondria	*H. maxima*	125	0.399	**0.744**	0.045	0.037	M	4	12	MT560546
nuclear-encoded *rps14* for mitochondria	*H. maxima*	155	0.170	**0.740**	0.041	0.041	M	3	20	MT560547

nuclear-	*H. asiatica*	150	0.031	**0.885**	0.014	0.182	M	2	7	MT560549
encoded	*H. henryi*	150	0.025	**0.895**	0.013	0.187	M	2	7	MT560548
rps16	*H. insularis*	150	0.031	**0.885**	0.014	0.182	M	2	7	MT560550
for plastids	*H. maxima*	150	0.031	**0.885**	0.014	0.182	M	2	7	MT560551
	H. nobilis var. *japonica*	150	0.031	**0.885**	0.014	0.182	M	2	7	MT560552
	Anemone narcissiflora	154	0.020	**0.881**	0.014	0.220	M	2	7	MT560553
	Pulsatilla koreana	154	0.023	**0.891**	0.016	0.203	M	2	7	MT560554
nuclear-	*H. asiatica*	163	0.016	**0.871**	0.009	0.225	M	2	7	MT560556
encoded	*H. henryi*	135	0.015	**0.888**	0.010	0.221	M	2	7	MT560557
rps16	*H. insularis*	149	0.017	**0.872**	0.009	0.224	M	2	7	MT560559
for mitochondria	*H. maxima*	150	0.015	**0.888**	0.010	0.221	M	2	7	MT560558
	H. nobilis var. *japonica*	118	0.022	**0.870**	0.015	0.165	M	2	7	MT560560
	Anemone narcissiflora	136	0.017	**0.875**	0.010	0.212	M	2	7	MT560555
	Pulsatilla koreana	135	0.019	**0.897**	0.010	0.163	M	2	7	MT560561

Supplementary Table S7. General characteristics of nuclear orthologs.

	A. narcissiflora	*H. asiatica*	*H. henryi*	*H. insularis*	*H. maxima*	*H. nobilis* var. *japonica*	*P. koreana*
Numer of unigenes by Trinity	151,868	106,836	181,489	109,522	112,733	123,856	91,319
TransRate score	0.1114	0.1374	0.087	0.1094	0.0281	0.1248	0.0889
Filtered good transcripts by TransRate	92,812	74,200	119,300	76,764	33,865	89,778	59,163
Number of complete ORFs	9,404	10,020	10,852	10,168	8,914	11,704	8,232
Number of genes in orthogroups	8,327	9,423	9,461	9,512	8,258	10,918	7,651
Number of orthogroups				11,456			
Number of orthogroups with all species present				1,428			
Number of single-copy orthogroups				510			

Supplementary Table S8. Scores of each criterion calculated by TreSpEx. Bold values indicate high degrees of misleading signals. Standard deviation and the average of upper quartile of long-branch scores, and the saturation degree was determined by slopes and R2 values of linear regressions of patristic distances against uncorrected distances p, with alignments of the 510 gene sequences.

Gene	Slope	R2	LB_score_upper_quartile	LB_score_Heterogeneity
OG0002494	**0.545768471**	0.98826928	15.7415164	14.59278659
OG0002036	0.820745464	0.997598744	20.66624549	17.75957658
OG0001963	0.671741599	0.96453961	32.16379982	23.8689893
OG0002718	**0.397374767**	**0.833014206**	32.9053919	27.98301341
OG0002711	0.589113358	**0.867415612**	36.39636745	33.10752059
OG0001931	0.691438822	0.988119374	48.56182057	34.90100371
OG0002260	0.754920914	0.991657836	50.92923091	35.95188417
OG0002000	0.818446973	0.994057758	53.70085616	36.86363922
OG0002510	0.765254458	0.98500621	49.81475954	37.03254655
OG0002631	0.727256407	0.988951573	49.93383298	37.46419116
OG0002564	**0.422920578**	**0.699239755**	41.29707122	37.84679462
OG0001933	0.842509471	**0.954329906**	51.22008315	37.86892097
OG0002605	0.631541082	**0.752212619**	48.42732332	38.04247379
OG0002560	0.702074349	**0.957845551**	47.74545886	38.74675972
OG0001838	0.882754524	0.995596721	55.04756092	39.52863227
OG0001947	0.646111524	**0.896038324**	49.0992213	41.01723792
OG0001986	0.811025991	**0.936140837**	52.41995889	41.64915763
OG0001960	**0.260823163**	**0.633953201**	48.22420843	42.46683224
OG0001984	0.833138507	0.994272516	61.99459112	43.44555263

OG0002302	0.705869784	0.978611622	61.22206736	44.4290584
OG0002543	**0.076584618**	**0.497789024**	65.03081544	44.50310725
OG0002719	0.69286654	**0.900461003**	52.61019885	44.93388709
OG0002465	0.874886899	0.998074966	62.50602716	44.95670793
OG0002187	0.743138739	0.995095345	64.47029513	45.28578079
OG0001880	**0.551845767**	**0.728412658**	50.42903671	45.29001316
OG0002052	0.903709011	0.998323707	65.92597846	45.4246741
OG0002402	0.747044665	0.992210815	63.21218862	45.60131959
OG0001834	0.789089739	0.997297163	66.26348478	45.60879292
OG0002626	0.810393814	**0.9424684**	62.66524594	45.64378728
OG0002700	0.644923496	**0.923332294**	52.22799047	45.83507343
OG0002378	**0.511249973**	**0.919382165**	67.18337267	45.92472502
OG0002628	0.796829829	0.997542784	65.43394083	46.16354194
OG0002380	0.886207572	0.998329688	64.59531038	46.28471801
OG0002562	0.670145556	**0.835495913**	62.44628628	46.40723413
OG0002447	0.703244178	0.98620912	66.25877304	46.45854824
OG0002575	0.756266991	0.989190362	67.95319992	46.82511657
OG0001907	0.879916086	0.99535076	69.24857237	47.49274437
OG0002645	0.663296801	**0.949506192**	60.65054789	47.57602505
OG0002388	0.772546676	0.970758415	68.32799133	48.00210009
OG0002445	0.726962582	0.974841209	69.37231876	48.09232372
OG0002701	0.924626225	0.999661985	68.3713958	48.14489103
OG0002027	**0.427717155**	0.990356182	70.13460496	48.30676703
OG0002587	0.794680268	0.997970429	71.09045779	48.65056871
OG0002049	**0.473513054**	**0.671224932**	65.06701333	48.6779521
OG0002223	0.879428362	0.995107957	69.54705493	48.68706367

OG0001964	0.774784275	0.996916204	71.18926138	48.81922686
OG0002086	0.877830804	0.98759904	70.07665752	49.1645656
OG0002591	0.808291445	0.990220158	70.93269853	49.21395441
OG0001943	0.724682205	0.989413534	71.46323777	49.24204002
OG0002430	0.658035554	0.994476508	71.88435377	49.51483228
OG0002593	0.783985474	0.996545819	71.48282116	49.55528847
OG0001831	0.79366358	0.996931655	71.42173005	49.59029745
OG0002606	0.783731188	0.994550746	70.35340452	49.60858307
OG0002148	**0.042073663**	**0.947983843**	72.74101602	49.70255259
OG0002394	**0.10708108**	**0.706593548**	73.15647145	50.07460723
OG0002136	0.851343493	0.997333141	71.35891325	50.08290613
OG0002288	**0.36752201**	**0.701446061**	67.45632956	50.15307449
OG0002171	0.687041296	**0.778684309**	72.8391521	50.23828539
OG0002295	0.650574489	0.979424346	73.37557437	50.30497868
OG0002503	0.892450714	0.998704654	70.33383913	50.32969331
OG0002600	0.827052497	0.998515307	73.26005111	50.33174105
OG0002454	0.66110199	0.985281651	71.52337598	50.5762262
OG0002315	0.733286164	0.975224137	73.71387994	50.6053376
OG0002045	0.779978221	0.996996683	73.39743236	50.66600404
OG0001969	0.928519679	0.999746482	73.29834124	50.77086783
OG0002589	0.918784875	0.998633473	73.02245397	50.77670201
OG0002521	0.775826966	0.996657798	74.51545833	50.98645598
OG0002670	0.808483297	0.996611811	73.09662912	51.02193002
OG0002616	0.908229831	0.996610831	72.87884659	51.03196554
OG0002576	0.740822459	**0.90210173**	73.43428323	51.0387014
OG0002630	0.844536552	0.998391018	72.16272591	51.0710339

OG0002705	0.738566032	0.995809556	73.27544282	51.12939893
OG0001828	0.741626572	0.997373084	74.7964128	51.27268977
OG0002145	0.730137302	0.995650935	73.94616588	51.35878202
OG0002343	0.78034726	0.994830441	74.74889754	51.55028433
OG0002710	0.810089359	0.99560444	73.66735624	51.61175787
OG0001957	0.877982154	0.998462667	72.65550487	51.62246079
OG0002029	0.894992408	0.999239776	73.45897281	51.66476135
OG0001847	0.766957517	0.996221996	75.11914514	51.82562567
OG0002624	0.918736494	0.999326719	73.75600168	51.82665175
OG0002400	0.892680672	0.999165438	74.52487651	51.84036675
OG0002681	0.908633553	0.998736499	75.8222795	51.88027083
OG0002595	0.810667444	**0.959471179**	75.16355319	51.90170659
OG0002275	0.882561883	0.999449465	74.46430908	51.98430861
OG0002274	0.796135094	0.998919815	75.71480534	52.09556753
OG0002309	0.856967433	0.998730332	76.14737737	52.17697077
OG0002601	0.814256334	**0.9356072**	74.25284185	52.17814147
OG0002131	0.616081015	**0.716712626**	75.79785503	52.21333211
OG0002635	0.876054861	0.999346985	73.48903342	52.26841688
OG0002150	0.707976076	0.992464006	74.08191202	52.28775664
OG0002189	0.727779026	0.997831473	75.90471547	52.2914593
OG0002384	0.921616276	0.99985413	76.2300887	52.30877147
OG0001946	0.642116896	**0.867369597**	76.43801261	52.43177239
OG0002632	0.769544077	0.998327965	75.87825593	52.43984488
OG0002396	0.905750171	0.99929456	75.94735641	52.47136062
OG0002128	0.872521073	0.999104166	76.59558469	52.5362885
OG0002137	0.902098216	0.999021031	74.97197379	52.55977899

OG0002186	0.874323536	0.997961098	76.75074866	52.57304028
OG0002448	0.825965749	0.998793482	76.25685702	52.59533219
OG0001835	0.802952363	0.991123909	72.85273957	52.63948521
OG0002111	0.799848817	0.998922389	74.50649632	52.72923411
OG0002642	0.870703938	0.99961559	76.76702248	52.83469646
OG0001917	0.951717999	0.999361799	77.09801087	52.94366328
OG0002340	0.792079264	0.994631509	73.75847846	53.01497353
OG0002184	0.878402861	0.998365691	77.47546038	53.11229664
OG0002499	0.884602165	0.999149211	77.33562244	53.20000573
OG0002081	0.808974142	0.998845896	76.82171048	53.24437157
OG0002181	0.82436577	0.998192367	78.03924979	53.50530247
OG0002078	0.852733854	0.998501501	76.91513551	53.50800106
OG0002296	0.843662097	0.998466051	78.28174362	53.52206965
OG0002346	0.827661782	0.994028842	75.27249676	53.63637023
OG0002354	0.932804923	0.999530554	77.43323094	53.6514233
OG0002013	0.776584825	0.999061074	78.39588109	53.66618801
OG0002090	0.827370736	0.998419023	77.57653832	53.67096612
OG0002122	0.801102048	0.998130757	77.71685339	53.67430857
OG0001905	0.787635613	0.996323306	76.55571619	53.74804554
OG0001849	0.633972764	**0.953588024**	77.87579003	53.77356481
OG0002014	0.896702732	0.998346708	77.77341098	53.82343751
OG0002639	0.68238743	0.987828447	76.44001248	53.86454473
OG0002066	0.810778414	0.999190444	78.46370553	53.87699135
OG0002266	0.759459466	0.996114242	78.59533823	53.90504412
OG0002650	0.802096682	0.99457902	78.29355827	53.94294272
OG0002089	0.800521382	**0.940088617**	77.78435056	53.9697023

OG0001993	0.704231683	0.989120564	70.50423453	53.9822513
OG0002508	**0.451543495**	**0.704333066**	78.79719161	54.00951659
OG0002696	0.71441319	0.996667742	78.09968018	54.01769628
OG0002578	0.778136956	0.996530723	78.85884231	54.03709278
OG0002571	0.911366921	0.999697277	78.73918546	54.05647969
OG0002625	0.76477028	0.968089508	78.71661108	54.06658317
OG0002016	0.759191359	0.99822208	79.06418045	54.152717
OG0002167	0.895236288	0.999092369	79.02283658	54.19681378
OG0002405	0.714227025	0.974754559	76.42509737	54.24033126
OG0002087	0.86225608	0.999069231	78.46283312	54.2761955
OG0002314	0.580441981	0.981214659	79.46275511	54.30256517
OG0002376	0.888758469	0.999557926	78.82985707	54.30867432
OG0001886	0.804779031	0.998507783	79.09403847	54.31886953
OG0002210	0.781601406	0.997501571	79.01532127	54.3297544
OG0002099	0.837608085	0.998731851	78.65850464	54.37555478
OG0002477	0.633578761	0.968035658	79.41020522	54.45940157
OG0002683	0.904405112	0.998822346	77.92587611	54.46391423
OG0001889	0.711856698	0.998050991	78.70957214	54.47593512
OG0002190	0.758338211	0.971852329	77.20989699	54.48457461
OG0001895	0.715337547	**0.853941063**	79.52926886	54.48963882
OG0002028	0.705397287	0.997199331	79.66682508	54.5430956
OG0002263	**0.302484644**	**0.573110667**	71.91493747	54.62706765
OG0002092	0.744437567	0.997616705	78.38411643	54.67322329
OG0002125	0.85530654	0.999356307	79.85621412	54.69562866
OG0002581	0.744206242	0.995252616	79.40481971	54.71501848
OG0001961	0.77060725	0.998083099	79.68047199	54.75265219

OG0002365	0.677900793	0.996283007	79.44129423	54.76910872
OG0002072	0.81883243	0.99555166	79.13262733	54.81815114
OG0002428	0.834546149	0.998045407	80.02565896	54.82106624
OG0002048	0.645992634	0.986438296	76.54088318	54.82167231
OG0002577	0.901476912	0.999856663	79.89588714	54.84627071
OG0001884	0.924942823	0.999124606	79.21593702	54.89801139
OG0002476	0.837054619	0.998641183	79.82171028	54.99755092
OG0002524	0.586166072	**0.934317132**	71.15197637	55.00438618
OG0002520	0.800855221	**0.894168041**	80.24689046	55.00999287
OG0001888	0.792692847	0.995526881	80.29596838	55.04672916
OG0002056	0.63424918	0.975012634	78.39349413	55.05267227
OG0002284	0.925113966	0.998790624	79.81168616	55.05562181
OG0002075	0.852179098	0.989329523	79.61314182	55.07020149
OG0002397	0.899164839	0.999059922	79.37460491	55.1155518
OG0002093	0.866390624	0.999748532	80.33631044	55.14772154
OG0002654	0.838885804	0.998853014	80.40921264	55.18264316
OG0002436	0.912093196	0.999045224	80.51979387	55.20637629
OG0002694	0.943307854	0.999787471	80.04519517	55.229051
OG0002331	0.852262288	0.99730053	79.35300778	55.23343559
OG0002351	0.839135539	0.999252513	80.20353017	55.24479238
OG0002425	0.738029849	**0.955026354**	80.68163586	55.27902819
OG0002168	0.769288921	0.997939112	80.09397032	55.29064819
OG0001967	0.759010519	0.996982253	79.37770635	55.29714487
OG0002480	0.886696574	0.999439758	80.49928439	55.38049774
OG0002297	0.777677483	0.99582381	80.37832841	55.40654461
OG0002366	0.775830767	0.997492724	79.97110569	55.4136279

OG0002697	0.772455898	0.999199564	80.74103613	55.42515454
OG0002196	0.773948955	0.998003248	80.82187951	55.42916542
OG0002272	0.71998211	0.996963277	80.78479647	55.43733978
OG0001971	0.735442481	0.965562157	80.65917268	55.44419739
OG0001909	0.757786223	0.998965364	79.47530784	55.46429133
OG0002594	0.925325588	0.99915364	79.88607583	55.46674983
OG0001837	0.844386957	0.996296726	80.84050221	55.47086059
OG0002258	0.777995814	0.998188403	80.24095811	55.52745482
OG0002094	0.763602001	0.998660561	81.17884493	55.5389107
OG0002656	0.924930745	0.999071868	80.51803858	55.60347618
OG0002144	0.867375729	0.997719001	81.1801303	55.6270901
OG0001918	0.748442248	0.999060713	80.7104339	55.63890684
OG0002061	**0.538582063**	**0.66257577**	81.33814756	55.65422671
OG0002134	0.809094132	0.980626095	81.33957405	55.66420833
OG0002505	0.906326567	0.99763975	81.05178674	55.66450113
OG0002511	0.878253476	0.99886037	81.24067253	55.69850214
OG0002130	0.831481481	0.998341844	80.40493403	55.71728755
OG0002313	0.833476132	0.998967334	81.49566857	55.72570969
OG0002493	0.80138335	0.996530299	81.15580157	55.74756552
OG0002126	0.609460595	0.981255896	81.23968476	55.78979084
OG0002434	0.76514428	0.998972018	81.16113291	55.80112407
OG0002671	0.832003944	0.995620902	79.61642782	55.80115771
OG0002404	0.906160992	0.999027215	81.65112056	55.83001261
OG0002002	0.855616482	0.996795804	78.46752594	55.83618276
OG0002201	0.931191378	0.999479236	81.71551028	55.83886024
OG0001856	0.835884543	0.998695707	79.90659775	55.87097941

OG0002500	0.688944953	0.993033476	81.50301582	55.90874562
OG0002381	0.711447071	0.996107107	80.82916668	55.98190326
OG0002475	0.711517143	0.99125807	81.23096496	56.01222182
OG0002247	0.809408247	0.998956408	81.96944345	56.02484803
OG0001987	0.908702928	0.999615803	81.87343694	56.03584009
OG0002653	0.818964072	0.99870205	81.87424134	56.0412683
OG0002088	0.739128787	**0.956267371**	78.23919019	56.05887764
OG0002177	0.912191393	0.999403201	81.89174995	56.06288191
OG0002255	0.692795643	0.990935259	80.96178952	56.1018982
OG0001923	0.857922489	0.998484968	81.36868017	56.13919303
OG0002460	0.79848341	0.98961769	81.79840872	56.14196982
OG0002254	0.819498636	0.998756588	81.44084476	56.16416565
OG0002479	0.827643969	0.999332252	81.15474541	56.16675589
OG0002422	0.78064435	0.996773605	81.12947231	56.17276418
OG0002335	0.783300729	0.984195745	81.4354387	56.17862307
OG0002393	0.808645654	0.996979674	81.01415094	56.18367506
OG0002127	0.89721876	0.998220205	77.55219652	56.19173326
OG0002300	0.857857817	0.999463574	81.78580925	56.23768425
OG0001830	0.740398313	0.991462865	78.66381336	56.26542723
OG0002620	0.824406048	0.998477261	82.08355828	56.28010262
OG0002285	0.897216803	0.997832111	81.05068761	56.29127313
OG0002246	0.597027325	0.994464925	82.39623749	56.32682745
OG0002133	0.726377847	0.996626725	82.06269418	56.35416773
OG0002147	0.844036436	0.998541224	81.75297651	56.37433646
OG0001891	0.801318284	0.999363276	82.10631172	56.42514192
OG0002466	0.717344606	0.9977236	82.3254048	56.43002423

OG0002234	**0.459513811**	**0.513911436**	82.03480053	56.44929704
OG0001914	0.676806876	0.993408542	82.40923115	56.45079114
OG0002588	0.762249124	0.971620222	82.1745424	56.45140007
OG0002648	0.841359825	0.995324338	82.28731236	56.46042403
OG0002484	0.800404909	0.997840914	82.3632442	56.46155337
OG0002586	0.819074529	0.998233701	82.14939111	56.55927449
OG0002514	0.623479983	**0.960869524**	82.5693486	56.6089849
OG0002264	0.854128504	0.997889054	82.63175074	56.61274691
OG0002668	0.737636174	**0.911103554**	81.37280329	56.6438604
OG0002046	0.883655505	0.999205327	81.95570637	56.65011786
OG0002022	0.811204244	0.99385696	81.1734554	56.70265937
OG0002228	0.915673695	0.999625836	82.83418546	56.73335523
OG0001877	0.771933357	0.998298052	81.90975368	56.77226855
OG0002252	0.812619972	0.998455695	82.63322649	56.78797929
OG0002237	0.78308151	0.99907042	82.59708653	56.83546803
OG0002079	0.83066893	0.989187572	83.2104418	56.85331895
OG0002429	**0.263060518**	**0.704857893**	71.75438557	56.89226346
OG0002159	0.777425749	0.995584307	82.98549632	56.92610441
OG0002598	0.704111295	0.997422899	82.2913677	56.93019429
OG0002032	0.913020715	0.99968385	82.67581054	56.97371925
OG0002708	0.877595531	0.998934674	83.08205583	57.06870625
OG0002371	0.857275295	0.998180104	81.6711403	57.089243
OG0002411	0.799644098	0.999553483	83.51425999	57.13305757
OG0002018	0.850216889	0.988247869	83.40701232	57.13547907
OG0002693	0.698669592	**0.95776148**	82.20623648	57.14883261
OG0002160	0.80825228	0.999107349	83.29329638	57.1526704

OG0002085	0.797695573	0.992557893	81.22636032	57.18771748
OG0002679	0.783673834	0.996038174	80.07823975	57.19167471
OG0002180	0.734298012	0.99542033	83.42197629	57.2105664
OG0002399	0.712588732	0.996407757	83.62392717	57.22513874
OG0002129	0.884935	0.999227487	83.43339259	57.24641064
OG0002157	0.702592493	0.968840197	81.43682336	57.26636344
OG0002407	0.809413984	0.998375321	82.7987155	57.30537095
OG0002435	0.784907417	0.997405521	81.73619278	57.342257
OG0002039	0.769169532	0.997545573	83.67352383	57.34280397
OG0002659	0.688797248	0.997551577	83.31087626	57.34627136
OG0001878	0.709399576	0.990788217	83.02623694	57.35303025
OG0001871	0.73633184	0.992806873	83.80075911	57.38200459
OG0002293	0.864950807	0.998492928	80.78235253	57.5079834
OG0002338	0.790966208	0.997597769	83.80106357	57.51939919
OG0001999	0.765693264	0.997337595	84.03021343	57.52664307
OG0002461	0.738125457	0.991826838	83.23353479	57.52756694
OG0002687	0.884673706	0.998887737	83.86265445	57.52871619
OG0002262	0.667093745	0.997891869	83.73186061	57.56383316
OG0001992	0.705967917	0.990962244	81.47716702	57.56802613
OG0001942	0.757898197	0.993433204	82.46747078	57.57767262
OG0002101	0.759975823	0.998380202	83.19157485	57.64604246
OG0002208	0.824053373	0.999234272	83.91630347	57.65417034
OG0002269	0.865203248	0.994523709	84.12371568	57.65836205
OG0002426	0.812241344	0.999120601	83.80915735	57.68756709
OG0002118	0.848306797	0.999297021	84.09430378	57.69650748
OG0002622	0.743164768	0.996368441	82.98394785	57.709068

OG0002355	0.647444114	**0.817368649**	84.01212871	57.72512463
OG0002379	0.838278343	0.999390971	83.60182599	57.73211993
OG0002279	0.837782412	0.99951407	83.78571585	57.74134024
OG0002248	0.74092378	0.99791044	83.40000285	57.77911252
OG0002103	0.889530014	0.99888725	84.38512848	57.79754009
OG0002421	0.774961676	0.994660102	84.6858204	57.90274812
OG0002542	0.758090631	0.996613349	84.53961826	57.91207332
OG0002690	0.887649786	0.998910913	82.88081736	57.91809325
OG0002170	0.841344812	0.999174064	84.41862313	57.94001122
OG0001962	0.837070683	0.998478715	82.9549027	57.96501081
OG0002412	0.899323469	0.998427407	84.56747624	57.96815779
OG0002619	0.876008583	0.999592262	84.44845296	57.97505632
OG0002304	0.775129599	0.994701282	83.19509952	58.03657857
OG0002173	0.73520377	0.994615584	84.68623007	58.06968157
OG0002283	**0.53617578**	**0.904704863**	83.41374484	58.12551179
OG0002050	0.72501307	**0.928694584**	84.30718652	58.13109408
OG0002330	0.916354391	0.999864121	84.79592381	58.13730293
OG0002215	0.725958805	0.991084738	85.17754194	58.21641269
OG0001869	0.795800591	0.997888429	84.99253847	58.23275646
OG0001919	0.883264692	0.999445683	81.75499919	58.25474425
OG0002033	0.871558455	0.99916441	80.45847026	58.25994535
OG0002415	0.668721861	0.99511328	85.03191273	58.27050665
OG0002373	0.875358874	0.996037538	83.90410246	58.34743476
OG0002240	0.800941977	0.99751752	84.08898055	58.3502476
OG0001893	0.797898855	0.998038251	84.9813754	58.35308445
OG0002389	0.862031463	0.998776702	81.30728942	58.43344813

OG0002392	0.650171183	0.990696508	85.262764	58.44788458
OG0002253	0.71149461	0.998011462	85.09447984	58.46304567
OG0002627	0.585902476	**0.875768977**	84.93611535	58.4712011
OG0002291	0.730965095	0.995894548	84.40317535	58.49405474
OG0002451	0.799962933	0.999039912	85.62850122	58.55517922
OG0002282	0.702445468	0.998294415	84.87456877	58.55846752
OG0002666	0.716485822	0.998095761	83.86213077	58.57105983
OG0002582	0.756517916	0.996361047	85.48846598	58.60497516
OG0001975	0.867612159	0.998604502	85.44121198	58.66278783
OG0002121	0.849208854	0.998408432	85.74209501	58.66543686
OG0001833	0.645896284	0.998099798	84.33309921	58.70171005
OG0002712	0.910499542	0.999490473	82.95986516	58.71628423
OG0001949	0.912396783	0.999269182	83.03546291	58.72634464
OG0002658	0.870276187	0.999246244	85.78126599	58.7443801
OG0002516	0.679325054	0.995295158	82.21567096	58.75908906
OG0002192	0.758786445	0.997851246	82.3542343	58.77689264
OG0002547	**0.467619366**	**0.701971177**	83.56320809	58.77936581
OG0002515	0.7773621	0.980187428	85.61397134	58.7878127
OG0002665	0.808790953	0.998488401	85.94169878	58.83281847
OG0002019	0.760296927	0.998476427	84.58542295	58.83369286
OG0002001	0.72475508	0.999376069	85.6256043	58.84873767
OG0002206	0.79444188	0.996241274	85.79987769	58.87359586
OG0002102	0.91671403	0.999773587	86.11516637	58.89382374
OG0002334	0.841589483	0.998955359	84.48957697	58.89733917
OG0002220	0.912079909	0.999680321	85.431137	58.91019172
OG0002676	0.807674056	0.992362376	85.87188526	58.9269377

OG0002146	**0.369106145**	**0.925441113**	79.03940201	58.93421825
OG0002444	0.906946822	0.999791868	85.97635549	58.9417644
OG0002664	0.753065547	0.997360461	85.93861207	58.94711223
OG0002059	0.690318745	0.991847001	86.1196543	58.97299948
OG0002156	0.904173517	0.999412246	85.87849157	58.98823684
OG0002572	0.804582748	0.9981685	85.12979406	58.99043441
OG0002009	0.744557849	0.997067584	86.20856951	58.99766897
OG0002390	0.750523674	0.994153076	85.20286598	59.00962383
OG0001958	0.815119633	0.997705892	83.50182562	59.01185589
OG0002583	0.878062309	0.999101624	83.46128654	59.0375335
OG0002257	0.864156436	0.999491115	85.54170285	59.05084234
OG0001920	0.768748724	0.99727217	86.17886479	59.05292255
OG0002348	0.644021313	0.994546165	84.62114382	59.05655669
OG0002212	0.664872662	0.99854728	86.2215799	59.11767946
OG0002485	0.707488546	0.994251456	81.74023217	59.11777103
OG0001904	0.718213046	0.990882987	83.13984607	59.12062562
OG0002164	0.733866331	0.994329307	85.7053142	59.129448
OG0002235	0.590083887	0.98879627	86.05326844	59.14204403
OG0002553	0.633741192	**0.924907919**	79.61701731	59.1904823
OG0002702	0.631566536	0.995484018	86.57519715	59.2134034
OG0002633	0.890186646	0.998170898	85.9972848	59.23488204
OG0002375	0.629916995	0.967141439	86.23871895	59.2506984
OG0002409	0.934408977	0.999866945	85.6881562	59.26910599
OG0001906	0.897787166	0.999657827	85.54198207	59.27511277
OG0001954	0.620469513	0.975929181	82.86308484	59.28880256
OG0001928	0.900232019	0.999642561	86.65541334	59.28981874

OG0002655	0.86112894	0.998893736	84.71022784	59.33919797
OG0002661	0.801578701	0.999476574	86.88004872	59.35835061
OG0002265	0.817837253	0.996529385	85.77349695	59.41622453
OG0002104	0.851410354	0.998876961	86.40226862	59.4236596
OG0002298	0.773135722	0.996126285	81.55418523	59.49992598
OG0002183	0.652263798	0.993886414	85.34526369	59.51362475
OG0002608	0.806038361	**0.884242664**	86.46933704	59.56748696
OG0002590	0.759023097	0.997724382	87.11918281	59.57807947
OG0002574	0.841732559	0.998070894	86.89250073	59.58769591
OG0001936	0.805589897	0.999343803	86.92941173	59.64019366
OG0002528	**0.125156372**	**0.166915194**	77.2584977	59.65358719
OG0002686	0.822162374	0.999411397	87.3030738	59.68626324
OG0002012	0.626742628	0.988859027	83.87955198	59.74109775
OG0002617	0.865279872	0.999083116	86.93396582	59.76181865
OG0002119	0.913155913	0.999415177	77.06701829	59.76616921
OG0002347	0.78103075	0.997339594	84.81492195	59.76977307
OG0002051	0.882350304	0.998959085	85.11910708	59.79704248
OG0002433	0.912192372	0.999829861	87.46692057	59.83540687
OG0001985	0.888670107	0.999256613	86.22576135	59.84601713
OG0002618	0.886078336	0.999188809	87.59421537	59.85716453
OG0002319	0.895497592	0.99964217	85.26894616	59.90173052
OG0002044	**0.493282249**	**0.82139057**	85.17092193	59.93026461
OG0002678	0.576047975	0.998974849	87.53429166	59.94753285
OG0001873	0.660270023	0.977090738	86.74402642	59.95389583
OG0002268	0.753754147	0.992941112	82.49423521	59.97541911
OG0002074	0.748876482	0.998665005	86.42373642	59.98934095

OG0002174	0.608596668	0.964517493	85.36838015	60.04510488
OG0002452	0.779628737	0.998730476	87.78472244	60.05516517
OG0002142	0.76441244	0.996246184	85.96294238	60.07273745
OG0002286	0.883551196	0.998435396	86.56512426	60.08962927
OG0001855	0.839603274	0.999323548	87.86854472	60.09395436
OG0002370	0.881161997	0.999861724	87.92475063	60.10814375
OG0002112	0.84134644	0.997830014	87.48045853	60.13272222
OG0002356	0.710177695	0.996268748	87.51742429	60.14315738
OG0002179	0.918915273	0.999211537	86.66246455	60.22337935
OG0002026	0.867350153	0.99919447	87.28875125	60.27056312
OG0002185	0.812120952	0.998096453	87.03659478	60.28534505
OG0002446	0.692564492	0.99482863	84.81293703	60.28693316
OG0001996	0.725097871	0.996883312	86.84365975	60.38725331
OG0002341	0.878521886	0.999351166	88.37807417	60.41178612
OG0002537	0.711873655	0.99435832	88.44361929	60.43144515
OG0002329	0.635412842	0.988035137	87.03474373	60.43998751
OG0001998	0.722919202	0.988618513	82.85966111	60.45463101
OG0001894	0.801075671	0.997988937	86.1013268	60.46604727
OG0002109	0.713965394	0.992606143	88.36607564	60.53623943
OG0001951	0.717749524	0.990569767	78.82367565	60.60780311
OG0002646	0.743195152	0.99726377	88.50593393	60.69137251
OG0002031	0.602656563	**0.933359709**	80.65153172	60.73185631
OG0002096	0.753963359	0.989728742	85.49576342	60.78320277
OG0002124	0.734747872	0.996573257	88.21205501	60.80675071
OG0002707	0.740097836	0.993761672	84.43697912	60.83372949
OG0001902	0.851574997	0.9987406	88.46049594	60.84520345

OG0001988	0.916409888	0.999257696	89.09044079	60.91134731
OG0002717	0.827817582	0.996523887	87.09566236	60.93517255
OG0002227	0.701481516	0.99246486	85.58045174	60.95861158
OG0002647	0.919138455	0.99980561	80.58559713	61.01064295
OG0002424	**0.554686818**	**0.907457946**	80.40523764	61.0298709
OG0002166	0.76579831	0.99903954	89.19793839	61.06989084
OG0001968	0.83277847	0.995264856	89.25976118	61.13941757
OG0002241	0.718058876	0.994511813	89.44471735	61.17755465
OG0002308	0.685749442	0.995167182	88.46616647	61.18552222
OG0002015	0.683575284	0.990479714	87.99322002	61.25044223
OG0002360	0.709306454	0.973171295	89.50126815	61.27159411
OG0002321	0.878944463	0.999788307	89.32975029	61.30236358
OG0002077	0.67800121	0.993432386	89.69249256	61.31218822
OG0002585	0.650887433	0.996691443	89.61295246	61.33588394
OG0001978	0.709032327	0.989687273	89.80596303	61.3548164
OG0002182	0.808067653	0.998987453	88.08466786	61.39019652
OG0002724	0.816026475	0.997279592	86.19931802	61.44005829
OG0001944	0.740339744	0.995668668	84.22385158	61.46657101
OG0001841	**0.511175874**	**0.838732849**	87.13818339	61.51828224
OG0001921	**0.272734571**	**0.504005645**	83.45677253	61.52140106
OG0002224	**0.557766257**	**0.954710792**	82.88908536	61.61028835
OG0002053	0.626860491	**0.939232834**	86.6206423	61.62884772
OG0002057	0.865249156	0.995902758	88.27086038	61.7002455
OG0002020	0.909611718	0.99908491	90.19399308	61.70665477
OG0002058	0.760962466	0.995851733	82.9145634	61.78367355
OG0001925	0.640852456	0.98971082	89.16174365	61.79246148

OG0002281	0.624844019	0.995484715	89.399243	61.7927011
OG0001952	0.896119274	0.999661883	88.74320019	61.98429821
OG0002276	0.798364657	0.999559984	90.31721194	62.03419138
OG0002155	**0.558821534**	**0.881992333**	86.67913782	62.21744641
OG0002214	0.705054854	0.993110951	86.71623306	62.24093689
OG0002259	0.717074988	0.99452343	82.55098594	62.2434114
OG0001872	0.763463576	0.999420851	90.22446758	62.25877468
OG0002496	0.873072551	0.999355363	90.54016996	62.28187666
OG0002175	0.796026333	0.996619754	85.29943988	62.39629526
OG0002685	0.920734615	0.999375603	89.75544375	62.42601539
OG0002490	0.679495405	0.977800935	89.28124566	62.47001252
OG0001956	0.574562473	0.97057927	88.58619792	62.6484658
OG0002482	0.606268474	0.994872386	91.82772934	62.85479841
OG0002387	0.678927289	0.991424452	89.96350755	62.86537538
OG0002063	0.91057147	0.999330189	89.41443004	62.90063907
OG0001994	0.856707482	0.998573944	87.46538904	62.92131992
OG0002199	0.914797083	0.9968542	90.08138379	62.97284975
OG0002226	0.655972145	0.994017454	89.90050459	63.03143836
OG0002233	0.629211172	0.985366081	85.09261807	63.03208766
OG0002561	**0.50431886**	**0.948365283**	90.72242194	63.29702412
OG0002579	0.65782096	0.992033407	90.38854904	63.37258959
OG0002083	0.697532823	0.986471764	85.56062453	63.54255341
OG0002249	0.651832049	0.989783818	87.07354934	63.61698242
OG0002527	0.723890711	0.991800139	90.38301232	63.72689867
OG0002229	**0.485176475**	**0.81155179**	92.46850718	63.74009518
OG0002084	0.657111083	0.992888459	90.7084087	63.87841945

OG0002080	**0.508748232**	0.984599766	91.5673035	63.88848226
OG0002292	0.698820278	0.99234435	93.1168581	63.90481856
OG0002420	0.746551898	0.99560309	90.64474242	63.95021533
OG0002573	0.886960993	0.999325208	93.35223958	64.23281393
OG0002673	0.674173254	0.987536941	86.47538865	64.39507711
OG0002525	**0.268442081**	**0.616341124**	71.0450817	64.40216884
OG0002369	0.76116533	0.997304366	90.49205377	64.47732357
OG0002172	0.724583295	0.995781109	89.50804152	64.73219169
OG0002037	0.907202299	0.999287752	92.05655153	64.87765906
OG0001940	0.94196283	0.999792184	94.63946494	65.15941169
OG0002603	0.676722833	0.990838543	91.10860956	65.7866929
OG0002038	0.922040431	0.999175716	90.6679321	66.08390762
OG0002568	**0.13067732**	**0.093295689**	92.66421298	66.11887491
OG0002097	0.950132865	0.999566525	89.51606624	66.17805328
OG0001995	0.662002915	0.99016124	93.31503247	66.23964379
OG0002188	0.794786406	0.996625437	86.43645505	66.67458022
OG0002613	0.738161557	0.996383414	93.85286192	66.74533619
OG0002326	0.578193457	0.98268267	**97.82274567**	**66.86826589**
OG0001899	0.934286623	0.999728392	**96.77433315**	**66.99125657**
OG0002312	0.685214421	0.99050879	90.7711198	**67.37509837**
OG0002294	0.825331384	0.997989031	91.82449947	**67.71138247**
OG0002143	0.641984889	0.984644761	95.43655242	**67.81032272**
OG0001887	**0.5143261**	**0.86233721**	**99.06338175**	**67.86387257**
OG0002431	0.760115684	0.987447927	93.2178304	**68.24762623**
OG0002008	0.751767604	0.991109452	94.91015648	**68.71395745**
OG0002723	0.688077902	0.979702279	91.27590115	**68.95134671**

OG0002280	**0.478639818**	**0.906296162**	89.16736236	**69.37351129**
OG0001900	0.689893397	0.985435974	**96.38902567**	**70.01633676**
OG0001989	0.849394652	0.998143322	94.90832401	**70.71080872**
OG0002011	**0.513000597**	**0.919977226**	**97.46594803**	**72.99190866**
OG0002140	0.746336735	0.995418567	**96.95703567**	**76.22013084**
OG0002243	**0.355747543**	**0.929216454**	85.83522748	**79.36373446**
OG0001836	**0.431439354**	**0.956756611**	**97.36787256**	**79.92744223**
OG0002660	**0.507174089**	0.980633852	93.55283131	**81.9718949**
OG0002599	**0.065413632**	**0.378178032**	85.79917205	**82.87929769**
OG0001934	**0.09690216**	**0.507641885**	85.8045383	**83.49563452**
OG0002726	**0.336528472**	0.968724218	87.32204755	**84.78110562**
OG0002054	**0.198600832**	**0.812742042**	93.6045791	**85.56434624**
OG0002449	**0.138868854**	**0.706646256**	88.59070257	**87.68587229**
OG0002691	**0.388805112**	0.98373882	91.21807609	**88.81985244**
OG0002095	0.728524485	0.998546377	**98.05794404**	**92.75618423**
OG0002025	**0.285835353**	**0.958362123**	**99.50505746**	**93.93541872**
OG0002244	**0.103573676**	**0.810147187**	**96.46397506**	**94.61056258**
OG0001972	**0.223403299**	**0.928428224**	**99.09640734**	**96.57713212**
OG0002035	**0.174225328**	**0.901869213**	**102.2427201**	**97.46274648**
OG0001991	**0.262722003**	0.973846619	**100.7131538**	**99.7416712**
OG0002592	**0.109420301**	0.972486848	**103.1878536**	**105.9758994**
OG0002607	**0.077309813**	0.963100086	**102.811395**	**106.0277268**
OG0002256	**0.015962588**	0.759433506	**102.7019782**	**107.5748154**

Supplementary Table S9. Model selection between species networks. Bold front indicates the optimal network.

Reticulartions	lnL	Branch lengths	*k*	AIC	△AIC
0	-1077.61188852455	8	8	2171.223777	74.23576904
1	-1049.99492872625	9	10	2119.989857	23.00184945
2	-1042.51504061667	10	12	2109.030081	12.04207323
3	-1036.65975450474	11	14	2101.319509	4.331501004
4	-1034.28901688722	12	16	2100.578034	3.590025769
**5**	**-1030.49400400281**	**13**	**18**	**2096.988008**	**0**
6	-1030.92900896427	14	20	2101.858018	4.870009923
